# A Modified ABCDE Model of Flowering in Orchids Based on Gene Expression Profiling Studies of the Moth Orchid *Phalaenopsis aphrodite*

**DOI:** 10.1371/journal.pone.0080462

**Published:** 2013-11-12

**Authors:** Chun-lin Su, Wan-Chieh Chen, Ann-Ying Lee, Chun-Yi Chen, Yao-Chien Alex Chang, Ya-Ting Chao, Ming-Che Shih

**Affiliations:** 1 Agricultural Biotechnology Research Center, Academia Sinica, Taipei, Taiwan; 2 Department of Horticulture and Landscape Architecture, National Taiwan University, Taipei, Taiwan; Michigan State University, United States of America

## Abstract

Previously we developed genomic resources for orchids, including transcriptomic analyses using next-generation sequencing techniques and construction of a web-based orchid genomic database. Here, we report a modified molecular model of flower development in the Orchidaceae based on functional analysis of gene expression profiles in *Phalaenopsis aphrodite* (a moth orchid) that revealed novel roles for the transcription factors involved in floral organ pattern formation. *Phalaenopsis* orchid floral organ-specific genes were identified by microarray analysis. Several critical transcription factors including *AP3*, *PI*, *AP1* and *AGL6*, displayed distinct spatial distribution patterns. Phylogenetic analysis of orchid MADS box genes was conducted to infer the evolutionary relationship among floral organ-specific genes. The results suggest that gene duplication MADS box genes in orchid may have resulted in their gaining novel functions during evolution. Based on these analyses, a modified model of orchid flowering was proposed. Comparison of the expression profiles of flowers of a peloric mutant and wild-type *Phalaenopsis* orchid further identified genes associated with lip morphology and peloric effects. Large scale investigation of gene expression profiles revealed that homeotic genes from the ABCDE model of flower development classes A and B in the *Phalaenopsis* orchid have novel functions due to evolutionary diversification, and display differential expression patterns.

## Introduction

Sexual propagation is an important physiological event for both animals and plants. In order to adapt to a dry environment, angiosperms have evolved many specialized flowering processes to achieve reproduction. Substantial work with model organisms ranging from morphological observation to study of molecular regulation has resulted in the elucidation of the reproductive development of flowering plants [1–3]. However, great diversity in reproductive behavior is observed in various plant taxa suggesting that alternative strategies have been adopted during the courses of evolution. Orchids are of particular interest to biologists because of their unique and intriguing biological traits. The Orchidaceae, one of the largest families of Angiosperms, displays a high degree of speciation [4] with wide variations in floral characteristics, including morphology, color, size and fragrance that ensure successful pollination. 

In contrast to most plant groups, which display radial symmetry in floral organ arrangement, orchids produce zygomorphic flowers [5]. The bilateral symmetry of zygomorphic flowers directs the approach of pollinating insects to the flowers from a particular orientation [6], and is thought to facilitate evolution and speciation [7]. The outer and inner perianth whorls of an orchid flower consist of two sets of three tepals. The outer tepals, also known as sepals, show colorful patterns similar to regular petals. One of the inner tepals, commonly referred to as a petal, is highly modified to form a labellum (lip), which has a distinct morphology and often serves as the landing platform for pollinator insects [5]. In addition, grains of pollen are aggregated to become pollinia that are attached to the top of the column [5]. The column is equivalent to a style and stigma that has an opening toward the labellum side. The stigma is inside the column.

The ABCDE model, which was modified from the original ABC model, describes the genetic mechanisms that establish floral organ identity in angiosperms (for reviews, see 8). Based mainly on studies in *Arabidopsis*, the ABCDE model explains flower development and maintenance of floral identity by an interactive network of transcription factors, especially MADS box factors. Many of these transcription factors were identified by mutant phenotypes and identification of the responsible alleles. For example, *APETALA1* (*AP1*) mutant, *ap1-1*, displayed a phenotype in which sepals became bract-like leaflets and petals were missing [9]. The *AP1* gene was demonstrated to interact with *LEAFY* genes in *Arabidopsis* to participate in the floral meristem formation [10]. *APELATA2* (*AP2*) loss-of-function mutants exhibited abnormal flower morphology with a partial carpel-sepal on the first whorl and semi-stamen in the petal layer and also abnormal seed development [11]. By the ABCDE model, floral identity-associated genes are divided into five functional classes. B functional class genes include two MADS box genes, *PISTILLATA* (PI) and *APETALA3* (*AP3*), which are expressed in petals and stamens but not sepals and pistils (whorl 2 and 3). *PI*/*AP3* defective mutants in *Arabidopsis* (*glo* and *def*) have abnormal development of the second- and third-whorl floral organs such as petals and stamens [12,13]. The AGAMOUS (AG) gene, which encodes a class C functional transcription factor, was discovered from T-DNA inserted *Arabidopsis ag* mutants, in which stamens and pistils were transformed into petals and sepals [14]. 

Besides *Arabidopsis*, several other plant species have been investigated to discover the mechanisms governing floral organ identity (for review, see 15). Snapdragon (*Antirrhinum* species) served as an early model plant in flowering studies due to its varied flower color and morphology. Transposable elements such as *Tam* and other molecular genetic tools helped to identify the genes responsible for polymorphic patterns among snapdragon flowers [16]. Transcription factors of functional groups in the ABC model were elucidated in studies of *Antirrhinum* [17,18]. In addition, *Petunia* species have been used to study the involvement of MADS box genes in flowering development including flower morphology, color and scent [19–21]. Columbine (*Aquilegia vulgaris*) was also proposed as a model organism to study the evolutionary mechanism driving adaptive radiation including changes in the length of the spur according to pollinators and habitats [22,23].

Despite a lack of relevant genomic resources and molecular tools, the orchid family has been proposed as a model group for studies of evolutionary innovation in floral diversity [24]. Functional genomic approaches have been used to characterize orchid MADS box genes in several orchid species: next generation sequencing (Roche 454) was used with *Oncidium* Gower Ramsey [25–27], an EST approach was used with *Phalaenopsis equestris* [28,29], and differential expression profiling with quantitative PCR was used with *Orchis italica* [30]. The co-expression patterns of these orchid genes in relationship to the ABCDE model of flowering and putative functional roles of the MADS box genes have been summarized and discussed [24,31]. However, none of these studies satisfactorily explained how the identity of orchid-specific floral organs such as the lip and pollina were determined.

Recently, our group has focused on developing genomic resources for orchids. We first developed an informatics pipeline for *de novo* assembly and annotation of the transcriptomic sequences obtained by next-generation sequencing technology [32]. We then constructed a web database to archive and share the transcriptomic information. The Orchidstra database (URL: http://orchidstra.abrc.sinica.edu.tw/) was constructed with rich sequence information of *Phalaenopsis aphrodite* together with other orchid species [33]. The sequence information was used to design a customized orchid microarray. Here, we report the application of this microarray to study molecular events of floral organ development and identity in *P. aphrodite*.

## Materials and Methods

### Plant materials

The moth orchid, *Phalaenopsis aphrodite* Rchb.f., was collected from its original mountain habitat in Dawu, Taitung County in Taiwan and was kindly provided by Dr. Tsai-Mu Shen from National Chiayi University (Chiayi, Taiwan). Sogo Yukidian ‘V3’ (a popular tetraploid commercial *Phalaenopsis* hybrid) were purchased from I-Hsing Biotechnology (Chiayi, Taiwan). Four cultivars of *Phalaenopsis* normal and perolic mutant flowers were used in comparative pattern analysis, including *Phalaenopsis equestris* purchased from OX Orchids Farm (Tainan, Taiwan); P. Little Mary, P. Nobby’s Amy and Dtps. I-Hsing Helen were purchased from I-Hsing Biotechnology. Mature orchid plants were maintained in 22-27°C growth chambers under a 12-hour day/night cycle with regular irrigation and fertilization. 

Plant tissues collected included leaves, roots, young inflorescences, flower buds, and flowers in full bloom from *Phalaenopsis aphrodite* Rchb.f. Different flower parts such as sepal, petal, lip, pollinia, column, pedicel and different stages flowers were from Sogo Yukidian ‘V3’.

### RNA isolation

Orchid total RNA was isolated as described previously with minor modification [32,34]. Plant tissues were frozen and ground in liquid nitrogen. RNA was extracted by vortexing tissue powder with 5 to10 volumes (w/v) of extraction buffer (2% hexadecyltrimethylammonium bromide, 1% polyvinylpyrrolidone 40, 100 mM Tris-HCl pH 7.5, 20 mM EDTA, 2 M NaCl, 2% 2-mercaptoethanol) pre-warmed at 65°C. The homogenate was incubated at 65°C for 15 minutes during which it was mixed several times. The homogenate was centrifuged at 3,000 × g for 10 minutes at room temperature. The supernatant was extracted twice with an equal volume of chloroform:isoamyl alcohol (24:1, v/v) and centrifuged at 8,000 × g for 15 minutes. One-third volume of 8M lithium chloride was added to the aqueous phase to precipitate total RNA at -20°C overnight. The RNA pellet was harvested by centrifugation at 12,000 × g for 30 minutes at 4°C then washed with ice-cold 75% ethanol, and re-suspended in RNase-free water. RNA purity and concentration were determined by Nanodrop 2000 (Thermo Scientific, MA, USA) or Qubit (Invitrogen, CA, USA) measurement. The quality of the RNA was evaluated on an Agilent 2100 Bioanalyzer (Agilent, CA, USA).

### DNA microarray fabrication

Probes for the orchid microarray were designed against transcriptome contigs of *Phalaenopsis aphrodite* from the Orchidstria database [32]. In principle, one probe was designed for one transcript contig except for those contigs longer than 500 base pairs in length, for which two probes were designed, one on the 5′ half and the other on the 3′ half. An additional 4 probes for each of the common orchid viruses, *Cymbidium* mosaic virus (CymMV) and Odontoglossum ringspot virus (ORSV), were added to the array design for viral detection. EST probes (39,431) and orchid virus detection probes (8) were identified by eArray (http://www.genomics.agilent.com/article.jsp?crumbAction=push&pageId=1456) software (Agilent, CA, USA). In summary, the first version of the orchid array applied in this study had a total of 67,038 probes. Each probe was made duplicated and Agilent spike-in control probes were included. All chips were manufactured by Agilent SurePrint technology (SurePrint G3 Gene Expression Microarrays, custom array, Agilent, CA, USA) The oligonucleotide probes were 60-mer in length and printed on regular 25 mm x 75 mm glass slides by in situ synthesis. The first version of the array was in a 4 x 180K format (Agilent design ID:030949), and the second version of the array was later developed that had 42,973 probes in an 8 x 60K format (Agilent design ID:033620.) 

An additional 3,534 new probes not present in the first version of array were designed from 3,517 ESTs annotated by tBlastX, 349 non-coding RNAs and 8 endogenous housekeeping genes (for 5′/3′ ratio). 

### Probe preparation and hybridization

RNA amplification was performed on all RNA samples before hybridization by MessageAmp™ II aRNA Amplification Kit (Ambion, TX, USA) starting with 1 µg total RNA according to manufacturer’s protocol. The whole procedure included first strand cDNA synthesis, second strand cDNA synthesis and in vitro transcription for cRNA synthesis. Finally, 1.65 µg of amplified aminoallyl labeled cRNA was coupled with Alex Fluor 555 dye (Cy 3), and then the dye-coupled aRNA was fragmented. All microarray hybridizations were followed by the Agilent Hybridization procedure at 65°C for 17 hours. All chips were scanned by an Agilent Microarray Scanner, with the scan settings at the manufacturer’s recommendation.

### Array data analysis

**Figure 1:**
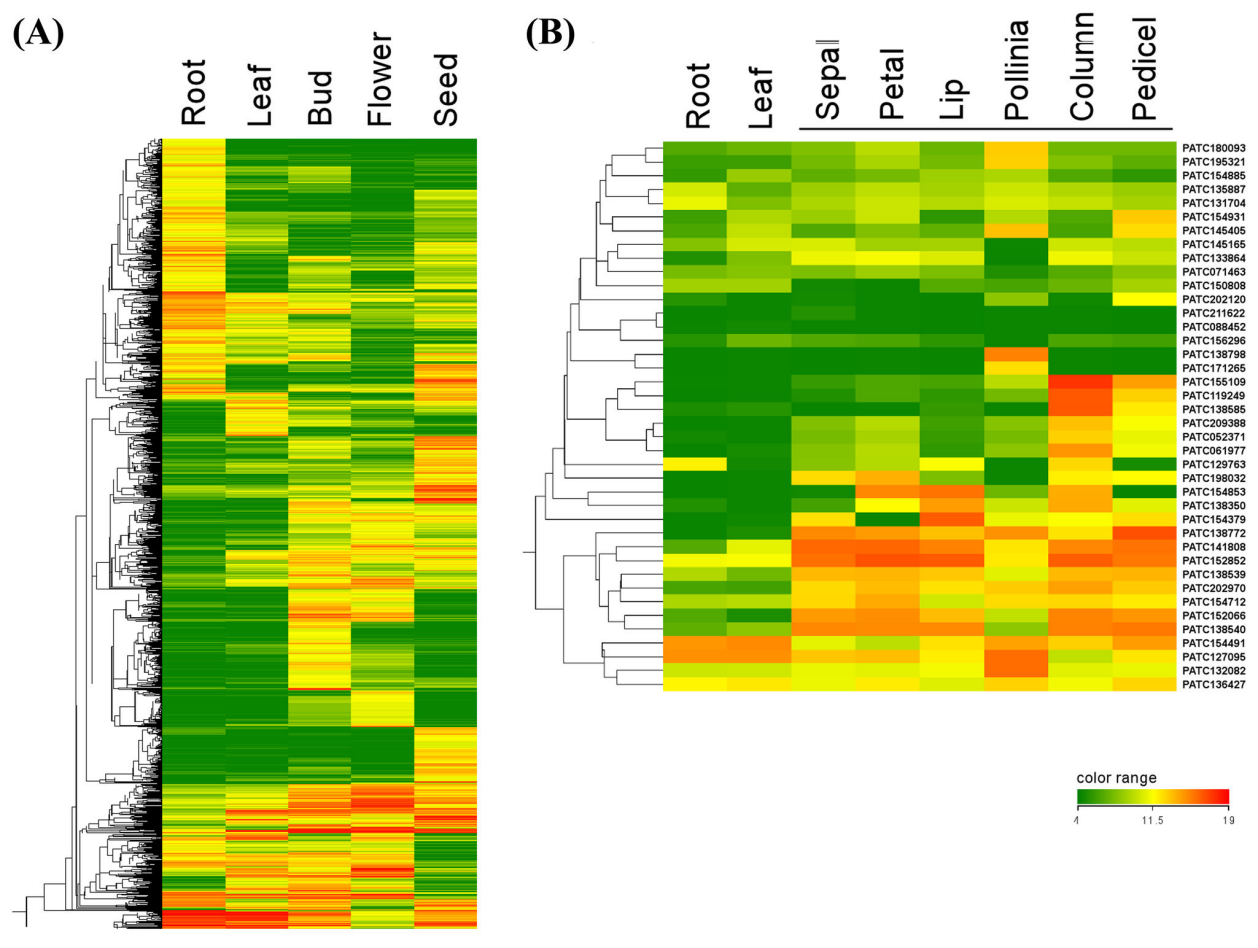
Gene clustering analyses of tissue-specific expression patterns in *Phalaenopsis* orchid. Genes that exhibited a greater than 4-fold change after microarray clustering analysis are shown. (A) Overall gene clustering of genes with greater than 4-fold change in expression was derived from a series of microarray analyses of a variety of *Phalaenopsis* tissues including root, leaf, flower bud, flower (full bloom) and germinating seed. (B) Gene clustering of *Phalaenopsis* orchid MADS box transcription factor encoding genes in various floral organ tissues. Only genes with levels of expression above the array detection in flower tissues are shown.

Tissues such as leaves, roots, flower buds, open flower and germinating seeds (Figure 1A) were repeated in triplicate in microarray experiments. Array experiments of the floral organs in Figure 1B were results of repeats from two collections of samples. All microarray data were preprocessed by Quantile normalization with a cutoff threshold of the raw signal at 20 in the GeneSpring GX11.5 package (Agilent Technologies, CA, USA). The normalized microarray data was submitted to the ‘Gene Expression Omnibus’ (GEO) database (http://www.ncbi.nlm.nih.gov/geo/, accession number GSE29910). 

### Validation of gene expression level using quantitative PCR

All the RNA samples were treated with DNase treatment by TURBO DNA-free kit (Ambion, TX, USA) and quantified by RNA Bioanalyzer (Agilent, CA, USA). cDNA was synthesized from 1 µg of total RNA with M-MLV Reverse transcrptase kit (Invitrogen, CA, USA) and polyT primer. All primers used were designed by Primer Express version 3.0 (Applied Biosystems, CA, USA). A total of 20μl real-time PCR reaction contained primers, cDNA and 10 µl 2X SYBR Green PCR master mix (Applied Biosystems, CA, USA). Real-time PCR was performed in the ABI Prism 7300 Sequence Detection System (Applied Biosystems, CA, USA) with programs recommended by the manufacturer (2 min at 50 °C, 10 min at 95 °C and 40 cycles of 95 °C for 15 sec and 60 °C for 1 min). Each sample was performed with real-time PCR for three independent biological replicates. The comparative C_T_ method (cycle of threshold) was used to determine the relative level of gene expression, with the expression value of ubiquitin (PATC150470) or actin (PATC135993) (PATC stands for *Phalaenopsis aphrodite* transcriptomic contigs used in the Orchidstra database, and denotes transcript contigs assembled from next generation sequencing data [33]), used as internal controls. Relative expression level is determined by delta C_T_ of target gene normalized to the internal control. Genes for the validation are listed in Table S2 in File S1 and their primers were listed in Table S3 in File S1.

### PCA and SPM analysis

Principal component analysis (PCA) by conditions and Gene Tree were both performed in GeneSpring GX11.5. Principal Component Analysis (PCA) is a dimension reduction method that has been widely used in gene expression analysis[35]. We use PCA to construct linear combinations of gene expressions that can represent the effects of all genes. PCA by conditions was performed using GeneSpring GX11.5 on a set of genes that were selected with expression above 500 in raw data, a p-value cut-off of 0.05 (Benjamini-Hochberg multiple testing correction) and a Lip/Petal fold change cut-off of 4.0. Gene trees were drawn by centroid-linkage hierarchical clustering with Euclidean distance used as similarity measure. Tissue specificity of each differentially expressed genes was also evaluated via SPM analysis [36] of microarray data. The range of the SPM is from 0 to 1. Higher SPM values indicate higher tissue specificity. We considered SPM > 0.8 as tissue specific expressed genes in this study. MapMan analysis was performed by identification of *Arabidopsis* homologs in the *Phalaenopsis* transcriptome which were then inputted into GeneSpring software or MapMan annotation tools from the MapMan web site (http://mapman.gabipd.org) [37].

### Phylogenetic analysis

The gene sequences used in the phylogenetic tree were downloaded from The Orchidstra Database (http://orchidstra.abrc.sinica.edu.tw/) or the NCBI GenBank (Table S4 in File S1). Full-length protein sequences or sequences of more than 100 amino acids were used. They were aligned by Mega5 [38] using the neighbor-joining method [39]. The topology of the phylogenetic tree was evaluated by analyzing 1000 bootstrap replicates of the data [40] and the evolutionary distances were computed using the Poisson-correction method.

## Results and Discussion

### Tissue-specific gene expression in orchid flower

To examine genome-wide gene expression profiles of orchids, a customized microarray chip was designed and constructed based on sequence information in the Orchidstra database [32] (http://orchidstra.abrc.sinica.edu.tw). Features and quality assessment of *Phalaenopsis* specialized microarray are described in Figure S1 in File S1.

An overview of microarray performance with gene clustering analysis of tissues including leaves, roots, flower buds at various stages, flower (open blossom), and germinating seeds of the *Phalaenopsis* orchid is shown in Figure 1A. Orchid flowers can be divided into six floral organs: sepal, petal, lip, pollinia, column and pedicel (comprising carpel and ovary). After performing microarrays of each floral part, fold-change analysis in GeneSpring v11.5 was used to identify floral organ-specific genes (Table S1 in File S1). Floral organ-specific genes were defined as those with normalized expression intensities at least 4-fold higher than those in the vegetative tissues. Alternatively, organ-specific genes were identified by the Specificity Measure (SPM) statistical method [36] after array data were normalized. SPM values range from 0 to 1, with a value close to 1 indicating high specificity as shown in Table S1 in File S1. Genes with organ-specific expression patterns were further validated by quantitative PCR (Figure S2 and Table S2 in File S1). 

### Flower-specific transcription factors in *P*. *aphrodite*

In our array data, members of transcription factor gene families, including MADS box, *bHLH*, *MYB* and *AP2* were found to exhibit organ-specific patterns. *KNOTTED1*-like homeobox (*KNOX*) family genes in other plant species have been reported to be flower-specific especially in the ovary [41] or flower and inflorescence [42]. Similarly, two *Phalaenopsis KNOX* genes, PATC145786 and PATC127065 (PATC stands for *Phalaenopsis aphrodite* transcriptomic contigs used in the Orchidstra database, and denotes transcript contigs assembled from next generation sequencing data [33]), were also identified to be flower- and/or inflorescence-specific by our orchid array analysis. Twenty-three of the 40 MADS box genes of *P. aphrodite* annotated in the Orchidstra database were differentially expressed in various flower organs (Figure 1B). In contrast, the expression levels of these MADS box genes were relatively low, sometimes undetectable, in vegetative tissues such as leaves or roots. Five MADS-box genes appear in the top 20 flower organ specific gene list with high SPM value (Table S1 in File S1). The expression patterns of these MADS box genes were diverse such as flower specific (*PaAGL6-2*), lip specific (*PaAGL6-1*), column specific (*PaAG-2*, *PaAG-3*) and pedicel specific (*PaAG-4*).

### Phylogenetic analysis of orchid MADS box genes

**Figure 2:**
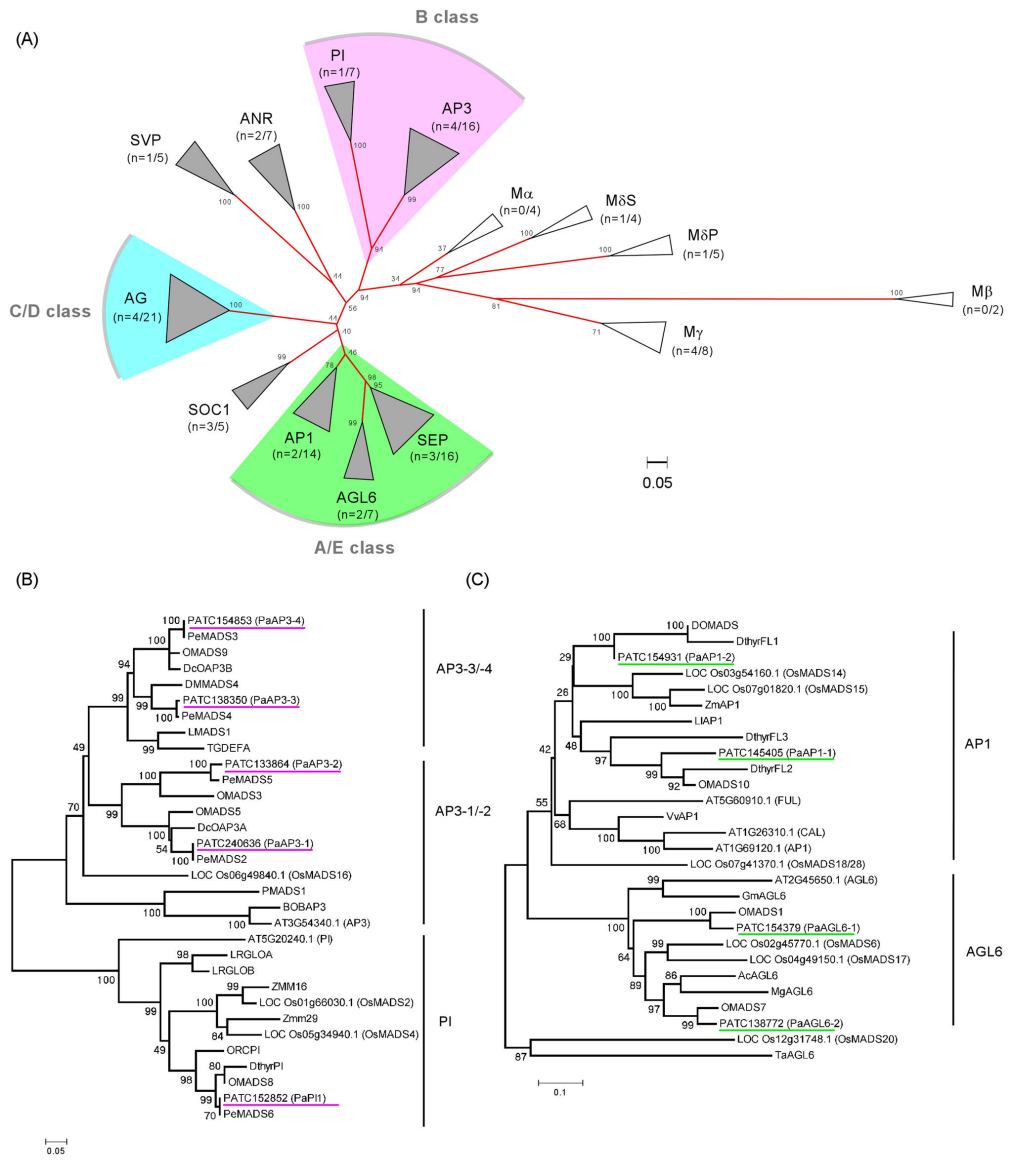
Phylogenetic analysis of the MADS box gene family. (A) A simplified view of groupings. MIKC-type MADS box genes including *APETALA3* (AP3), *PISTILLATA* (PI), *ANTHOCYANIDIN* *REDUCTASE* (ANR), SHORT VEGETATIVE PHASE (SVP), AGAMOUS (AG), *SUPPRESOR* *OF* *OVEREXPRESSION* *OF* *CONSTANS1* (SOC1), *APETALA1* (AP1), *AGAMOUS-LIKE6* (AGL6) and *SEPALLATA* (SEP) are indicated by unfilled triangles. M-type MADS box genes including *Mα*, *Mβ*, *M*γ and *M*δ(S type and P type) are indicated by unfilled triangles. The A/E class of MADS box gene comprises *AP1*, *AGL6* and *SEP* genes; the C/D class comprises of *AG* genes and the B class comprises of *PI* and *AP3* genes. The numbers in the parenthesis indicate the number of MADS box genes identified in *P*. *aphrodite* versus the total number of genes. Altogether, 121 MADS box genes were used in this phylogenetic analysis: 30 from *Arabidopsis*, 28 from rice, and 63 from orchid species (among which, 28 were identified in *P*. *aphrodite*). (B) Detailed phylogenetic analysis of class B genes, *AP3* and *PI*. (C) Detailed phylogenetic analysis of class A genes *AP1* and *AGL6*. Accession number and gene information are listed in Table S4 in File S1.

Since more than half of MADS box genes from *P. aphrodite* showed great specificity in floral organs, a phylogenetic analysis of orchid MADS box transcription factors was conducted. To date, 107 MADS box genes have been identified in *Arabidopsis*. These genes can be divided into 5 groups, MIKC, *Mα*, *Mβ*, *Mγ* and *Mδ* [43,44]. We retrieved 30 *Arabidopsis* and 28 rice representative MADS-box genes from GenBank to construct the mainframe of a phylogenetic tree. Twenty-eight MADS box genes in *P. aphrodite* (from the Orchidstra database) with sequences longer than 100 deduced amino acids, including those with flower-specific expression patterns (Figure 1B), were included in the phylogenetic analysis. Thirty-five MADS box genes in other orchid species, such as *Cymbidium*, *Dendrobium*, *Oncidium* and other *Phalaenopsis* species were retrieved from NCBI GenBank or the Orchidstra database. A brief version of the phylogenetic tree constructed is shown in Figure 2A. (For the detailed phylogenetic tree, see Figure S3A and the gene list in Table S4 in File S1). Genes from the *Mα* and *Mβ* groups have not been previously identified in orchids, probably because these genes have low expression levels under normal growth conditions. Compared to *Arabidopsis*, some of the MADS box genes in *Phalaenopsis* orchid have more copies, such as three *SEP* genes, three *SOC* genes, four *Mγ* genes, four *AP3* genes and four *AG* genes. Other sub-groups of MADS box genes comprising only one or two genes were also identified in the *Phalaenopsis* transcriptome (Figure 2A and Figure S3A in File S1).

Our microarray analyses identified 23 flower-specific MADS box genes in *P. aphrodite* (Figure 1B). Inspection of the phylogenetic trees in Figure 2 showed that 16 of these 23 MADS box genes were grouped together with different functional classes of floral identity genes in *Arabidopsis*. We therefore renamed these 16 genes based on their functional classes. Table 1 illustrates the functional grouping of these genes and their corresponding contig numbers in the Orchidstra database. 

**Table 1:** ABCDE Functional Classes of MADS-Box Genes in *Phalaenopsis aphrodite*.

**Class**	**Clade**	**Name**	**Orchidstra Accession Number**
**B**	AP3	PaAP3-1	PATC240636
	AP3	PaAP3-2	PATC133864
	AP3	PaAP3-3	PATC138350
	AP3	PaAP3-4	PATC154853
	PI	PaPI-1	PATC152852
**A**	AP1	PaAP1-1	PATC145405
	AP1	PaAP1-2	PATC154931
	AGL6	PaAGL6-1	PATC154379
	AGL6	PaAGL6-2	PATC138772
**C/D**	AG	PaAG-1	PATC052371
	AG	PaAG-2	PATC138585
	AG	PaAG-3	PATC155109
	STK	PaAG-4	PATC202120
**E**	SEP	PaSEP-1	PATC138540
	SEP	PaSEP-2	PATC141808
	SEP	PaSEP-3	PATC152066

### Subcellular localization MADS box factors

The subcellular localization of several *Phalaenopsis* MADS box genes was examined by particle bombardment of green fluorescence protein fusion constructs into the orchid petal. Images from confocal microscopy of 20 *Phalaenopsis* MADS box proteins are shown in Figure S4 in File S1. Eighteen of the MADS box proteins exhibited nuclear localization patterns. Among these, *PaSEP-1* and *PaAGL6-2*, exhibited nucleolar localization, suggesting that their functions may be associated with ribosome biogenesis [45]. *PaAP3-1* and *PaPI-1* exhibited a nuclear speckle pattern, suggesting roles in transcription and splicing activities [46]. *PaAP3-4* and *PaSVP-1* exhibited both nucleic and cytoplasmic localization with *GFP*-fusion protein, indicating that these two factors may also have cytoplasmic regulatory functions in addition to being nucleic factors.

### Differential expression of MADS box genes

**Figure 3:**
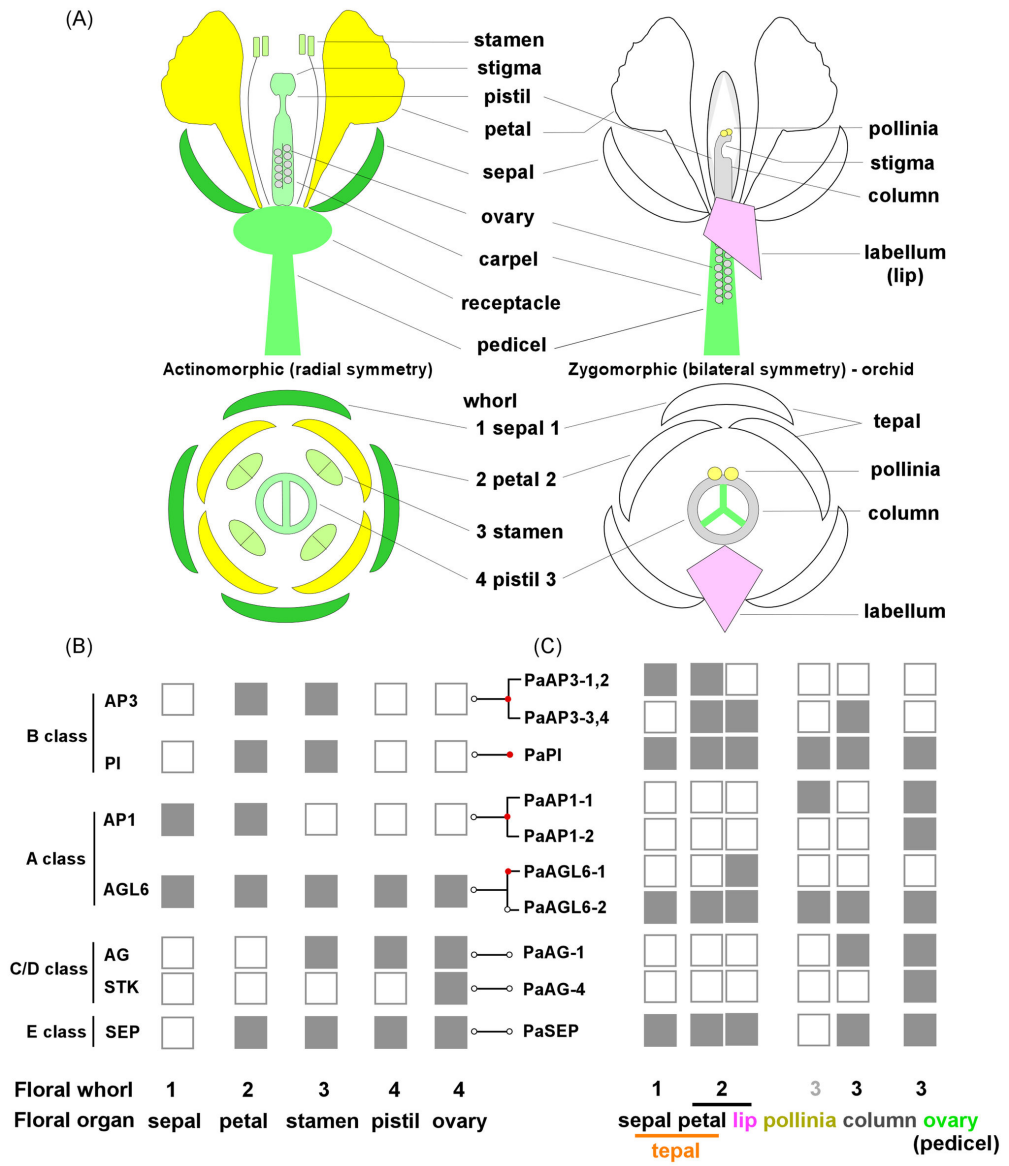
Comparative models of flower development in actnomorphic and zygomorphic angiosperms including morphological variation and distribution of transcription factors. (A) Arrangement of floral organs in radial symmetry as seen in most angiosperms and the bilateral symmetrical arrangement seen in orchid. Top views of both types of flowers show the arrangement of whorls of organs. The orchid flower lacks the stamen whorl and pollinia are attached to the apex of the column. (B) Flowering model showing classes of functional transcription factors discovered from studies of *Arabidopsis* [43,58,59]. (C) Flowering model showing transcription factors identified to be floral organ-specific in *P*. *aphrodite*. Solid boxes indicate the presence of gene expression and empty boxes indicate the absence of gene expression. The connecting line between panel C and panel B indicates phylogenetically related genes. Red dots indicate diversification of spatial distribution and open dots indicate conserved distribution.

**Figure 4:**
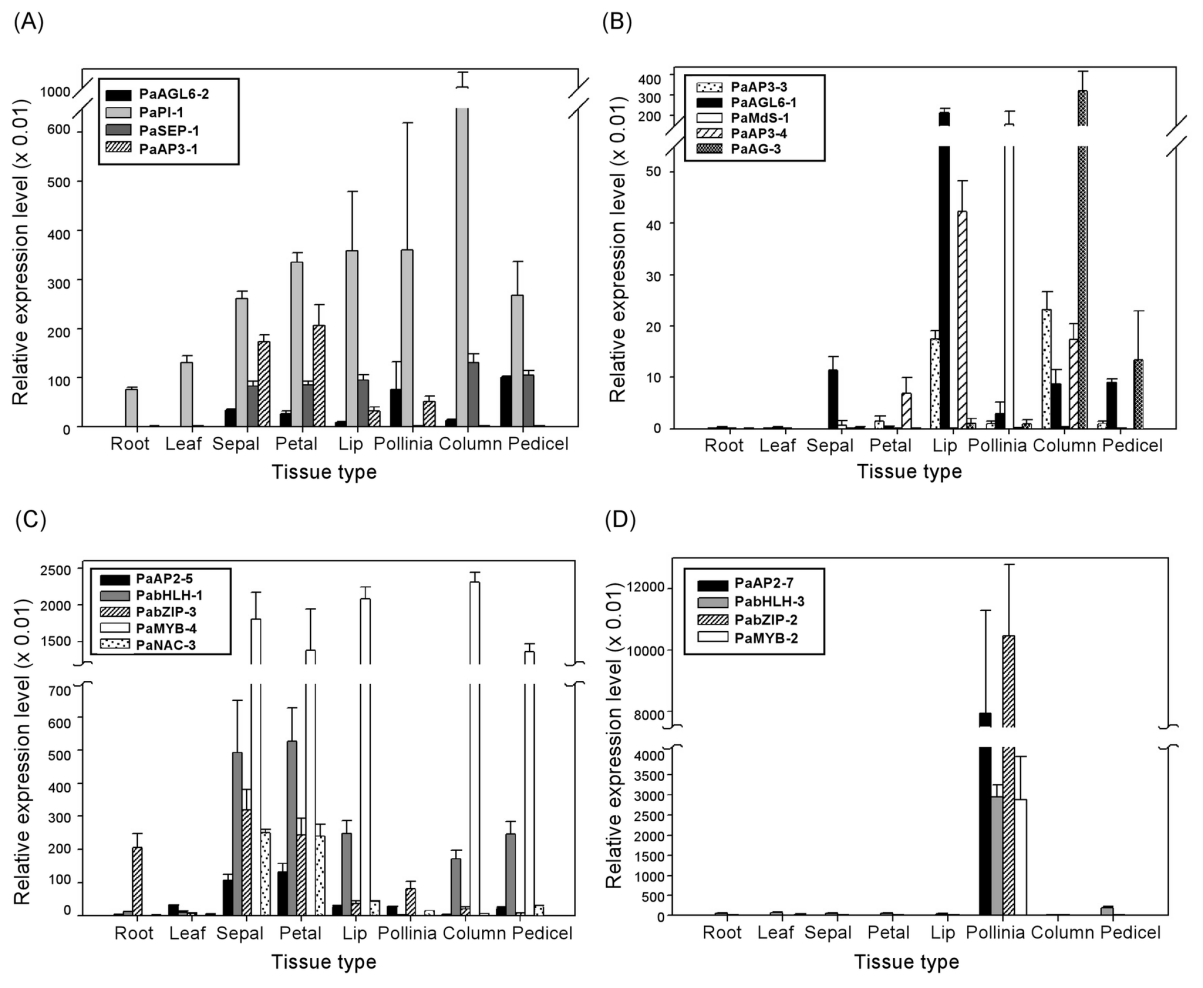
Quantitative PCR validation of orchid transcription factors with floral organ-specific expression patterns. (A) Relative expression levels of MADS box genes expressed across floral organs. (B) Relative expression levels of MADS box genes with floral organ-specific expression patterns. (C) Relative expression levels of *AP2*, *bHLH*, *bZIP* and *NAC* that are generally expressed across floral organs. (D) Relative expression levels of transcription factor genes with pollinia-specific expression patterns. Primers used in the PCR reactions are listed in Table S3 in File S1.

The morphological variation in the arrangement of floral organs in orchid flowers is unique among the angiosperms (Figure 3A). The zygomorphic (bilateral symmetric) arrangement of orchid floral organs together with the lack of stamen whorl (pollinia structure attached to a column), lip formation, and petalized sepals make orchid blossom unique among flowering plants.In *Phalaenopsis* orchid, five class B MADS box genes (four *AP3* and one *PI*) exhibited three expression patterns. The first pattern, represented by *PaAP3-1* was predominant expression in sepals and petals, whorls 1 and 2 (Figure 4A). The second pattern, represented by *PaAP3-4* and *PaAP3-3*, was high levels of expression in the lip and column (Figure 4B). The third pattern, represented by *PaPI-1* (Figure 4A) and *PaAP3-2* (Figure 1B), was high levels of expression in all flower organs except the pollinia. The expression patterns of *PaAP3* and *PaPI-1* genes are similar to those observed previously with *PeMADS* (2–5) in *Phalaenopsis equestris* and *OMADS* (5, 3, 9 and 8) in *Oncidium* orchid [27,28]. 

The expression patterns of four class A genes, *PaAGL6-1*, *PaAGL6-2*, *PaAP1-1* and *PaAP1-2*, were validated by RT-PCR and are summarized in Figure 3C *. PaAGL6-2* was expressed throughout all floral organs, which is similar to the expression pattern of *AtAGL6* in *Arabidopsis* [47]. In contrast, *PaAGL6-*1 was expressed specifically in the lip (Figure 3C). These results suggest that *PaAGL6-2* might have a similar role to *AtAGL6* and that *PaAGL6-1* might gain other function to contribute the formation of lip in orchids. In grasses (Poaceae), *AGL6* was found to be expressed in various floral organs such as ovules and lodicules as well as floral meristems and is thought to be pleiotropic having acquired multiple functions over its evolution [48]. Ectopically expressing *AGL6* homolog of *Oncidium* orchid (*OMADS1*) resulted in an early flowering phenotype as well as morphological changes in sepal and petal of *Arabidopsis* [49]. 

*PaAP1-1* was expressed mainly in the inner whorls of the pollinia and pedicel (Figure 3C), indicating that its function may deviate from the function of the class A genes that are required for maintaining the homeostasis of whorl 1 and 2 (sepal and petal). *PaAP1-2* was also specifically expressed in the pedicel. A similar result was observed in *Oncidium AP1* homolog, *OMADS10* [26]. Taken together, these results suggest that orchid *AP1* might be involved in promoting the development of the pollinia and gynoecium rather than being involved in perianth formation. 

The E class genes, *PaSEP1-1*, *PaSEP1-2* and *PaSEP1-3*, were highly expressed in all floral organs except the pollina (Figure 3C). These patterns were similar to the E class genes in *Arabidopsis*, except that the *AtSEP* genes were not expressed in the sepal. Similar to *Arabidopsis*, *Phalaenopsis* orchid has multiple copies of *PaSEP* genes with similar expression patterns and functional redundancy (Figure S3A in File S1) [50]. In a previous study of *P. equestris*, *PeMADS1* and *PeMADS7* were identified to be members of the class C/D functional group [29]. In *P. aphrodite*, several MADS box genes, designated as *PaAG* (*AGAMOUS*) were found to have expression patterns similar to those of C and D class MADS box genes. Among *PaAG* genes, *PaAG-1*, *PaAG-2* and *PaAG-3* were full length. These three genes together with PATC119249 and PATC209388 were annotated to encode AG-like proteins, but the amino acid sequences of PATC119249 and PATC209388 were too short for a clear phylogenetic classification. All five genes were highly expressed in the column and pedicel (see the expression patterns in Figure 1B and validation of *PaAG-3* in Figure 4B). *PaAG-1*, *PaAG-2* and *PaAG-3* were all demonstrated to be nucleic factors (Figure S4 in File S1). Expression profiling of the *PaAG* genes in orchid indicated that the functions of class C MADS box genes are duplicated and evolutionarily conserved. *PaAG-4* encodes an AG protein from class D, with specific expression in the pedicel (carpel and ovary) (Figures 1B and 3C).

### A modified ABCDE model of floral organ identity in orchids

The data presented here show that the gene expression profiles of C and D class MADS box genes in orchids are similar to those in *Arabidopsis* (Figure 3B and 3C), suggesting functional conservation of C and D class genes between these two species. In addition, the expression patterns of the E class *SEP* genes were similar in *P. aphrodite* and *Arabidopsis* except that expression of *PaSEP* was extended into the sepal where *AtSEP* was not expressed. In contrast, the expression patterns of orchid class A and B genes are more diversified than the conventional pattern. *PaAGL6-2* and *PaAp3-3* had expression patterns similar to their *Arabidopsis* homologs [12,47], but *PaAGL6-1*, *PaAP1-1*, *PaAP3-1* and *PaSep* had distinct expression. *PaAGL6-1* was only expressed in the lip and *PaAP1-1* was no longer expressed in the outer whorls. *PaAGL6-1* is homologous to *Oncidium OMADS1* [49] and may be specialized from monocot *AGL* genes for a unique role in lip formation (Figure 2C). The class B genes, *PaAP3-1* and *PaPI-1*, extend their expression into the sepal. Inspection of the phylogenetic tree (Figure 2) suggested that *PaAP3* may be derived from ancestral gene duplication to generate two sub-clades of *AP3* homologs [24,27,51], while *PaPI* seemed to continue the lineage but clustered to form an orchid-specific group (Figure 2B). In addition, *AP1* in the monocots behaved very similarly to *PaAP3* in the phylogenetic tree in which both evolved into two sub-clades while deviating from the dicot *AP1* clade (Figure 2C). This kind of evolutionary radiation may be responsible for the gain of novel functions for *PaAP3* and *PaAP1* genes in orchids. Taken together, the unique expression patterns and phylogenetic groupings strongly suggest that some of the orchid MADS box genes have evolved and become specialized into regulating different whorls, which were different from their counterparts in *Arabidopsis* and other model organisms whose study contributed to the formulation of the ABCDE model. We conclude that *PaAP3* and *PaSEP* genes extended their expression into the sepal resulting in its petal-like morphology in orchids. *PaAGL6-1* was specialized from the *AGL6* genes and plays a different role, promoting one of the petals to become the lip. *PaAP1* genes have evolved into two clades and gained novel functions by expressing in the inner whorls. 

In addition to *AGL6* and *AP1*, *AP2* is an essential class A functional gene in *Arabidopsis*. *AP2/ERF* is a large gene family of transcription factors, with 138 members in *Arabidopsis* [52]. There are 78 *Phalaenopsis AP2* genes identified in the transcriptome database, 38 of them with deduced amino acid sequences long enough for phylogenetic analysis (Figure S3B in File S1). Among the flower-specific *AP2* genes of *Phalaenopsis* orchid, *PaAP2-5* was expressed in the outer two whorls (sepal and petal) (Figure 4C). *PaAP2-7* was expressed with Pollinia-specific pattern (Figure 4D). *PaAP2-11* was expressed at a low level among all floral organs, similar to that of *Arabidopsis AP2* (AT4G36920) [11]. In petunia, three *AP2* homologs (*PhAp2*) were identified and *PhAp2A* was demonstrated to be the *Arabidopsis AP2* ortholog with similar expression pattern and was able to complement *Arabidopsis ap2-1* mutant. *PhAp2B* and *PhAp2C* exhibited less sequence homology and divergent expression patterns [53]. The diverse expression pattern of multiple orchid *AP2* genes suggests that they might have evolved and obtained different functional roles in the flower development. 

In order to construct a flowering model suited to orchids, the ABCDE model needs to be modified to explain the fact that orchids lack a stamen whorl. Many of the MADS box genes such as *PaAP3* and *PaSEP* described earlier were downregulated in the Pollinia. *PaMδS-1* (PATC138798) and *PaMδP-1* (PATC132082) are both *Mδ*-type MADS box genes that exhibit Pollinia-specific expression patterns (Figure 1B and 4B). In *Arabidopsis*, *MδS*- and *MδP*-type MADS box genes were highly expressed in pollen and when multiple *Mδ*alleles were defective, fertility was severely reduced due to low pollen viability [54]. Several other transcription factor genes, such as *PaAP2-7* (PATC138345), *PabHLH-3* (basic helix-loop-helix protein, PATC140443), *PabZIP-2* (basic leucine zipper protein, PATC134262) and *PaMYB-2* (PATC152106), also showed Pollinia-specific expression patterns (Figure 4D). The opposite expression pattern was observed for *PaAP2-5* (PATC133172), *PabHLH-1* (PATC135043), *PabZIP-3* (PATC136849), *PaMYB-4* (PATC150065) and *PaNAC-3* (*NAM*, *ATAF*, *CUC* proteins, PATC138298), which were expressed highly in all floral organs but not in the pollinia (Figure 4C). This kind of gene expression pattern is rather peculiar and has not been described in previous studies. More in-depth research needs to be conducted in order to understand the mechanism of pollinia pattern formation in orchids. 

### Comparative expression analysis of wild-type and peloric mutant flowers

**Figure 5:**
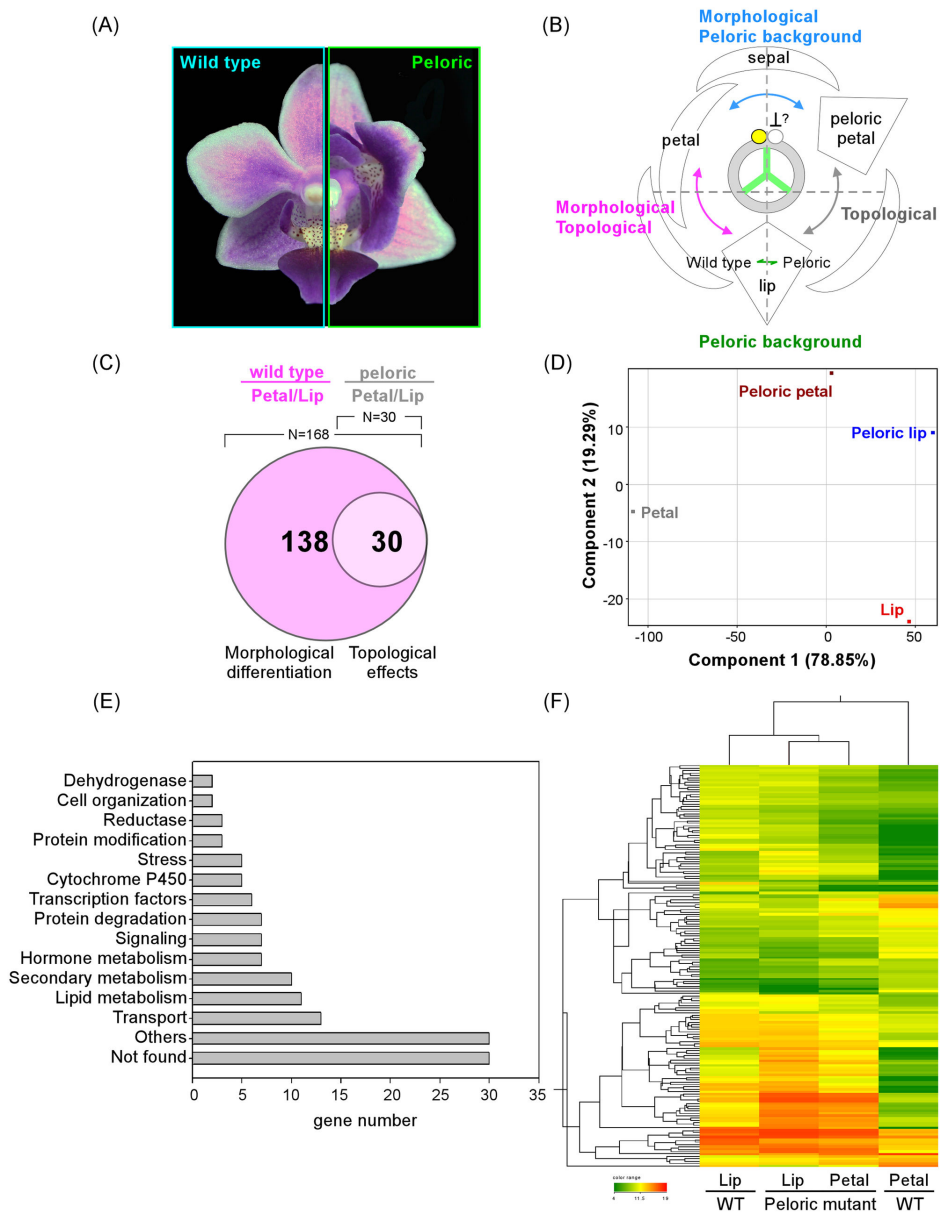
Gene classification from comparative analysis of expression profiles of wild-type and peloric mutants. (A) Phenotypes of wild-type and peloric mutants of *Phalaenopsis* *equestris*. (B) Proposed model of mechanism of peloric formation. (C) Venn diagram of genes differentially expressed in petal and lip tissues of either wild-type or peloric mutant (difference > 4-fold). (D) Principal component analysis (PCA) of genes shown in C that are differentially expressed in petal or lip tissues. (E) MapMan analysis of gene categories from PCA analysis (absolute value of loading score > 0.8) in D. (F) Genes clustering analysis of 168 genes in C that are differentially expressed in petal and lip tissues.

Mutants with polymorphic flower phenotypes provide useful tools in the investigation of transcription factor functions. For reasons as yet unknown, the two lateral petals in the orchid flower sometimes become lip-like due to a peloric mutation (Figure 5A, for more peloric phenotypes see Figure S5 in File S1). The peloric mutation transforms the bilateral symmetric orchid flower into radial symmetric structure by the formation of three lips in the petal whorl. The peloric mutant is often associated with defective development of the pollinia and column. Compared to the wild-type, expression of genes involved in DNA methylation and chromatin remodeling is increased in peloric mutants [55], suggesting that the peloric phenotype may be the result of epigenetic effects. In order to identify genes that are co-expressed in the lip structure and the genes responsible for peloric transformation, we compared the expression profiles of the petal and lip of flowers from wild-type and a peloric mutant of another moth orchid, *P. equestris*. Comparative profiling of wild-type and peloric flowers allowed genes involved in morphological, topological and peloric development to be grouped for more detailed studies (Figure 5B). Differential genes responsible for morphological differentiation can be identified by comparing petals of wild type to the peloric mutant (Figure 5C). Thirty genes that may be responsible for topological arrangement of the second whorl were deduced from comparing profiling of petal to lip whether in the wild type or in the mutant (Figure 5C). The Venn diagram shows the number of genes differentially expressed in the petal and lip in the wild-type and peloric mutant (Figure 5C). A total of 168 genes exhibited differential expression levels in the petal and lip in the wild type. Among these 168 genes, 138 genes that did not show a change between the peloric petal and peloric lip were postulated to be genes responsible for morphological differentiation. The remaining 30 genes that were differentially expressed between the petal and lip in the wild-type or peloric mutant, were postulated to be responsible for the topological arrangement of the second whorl (Figure 5C). The 168 differential genes from the petal/lip comparison were subjected to Principal Component Analysis (PCA) to identify organs that have similar gene expression patterns. The first principal component accounted for 79% of the variance in the selected genes, and the first and second principal component accounted for over 98%. The PCA plot (Figure 5D) revealed that peloric petals formed clusters with the lips by the genes input was projected onto the first component. In the positive correlation of component 1, genes with significant loading score value (absolute value of loading score > 0.8) were further categorized into functional groups through MapMan analysis (Figure 5E). Large group of genes in lipid metabolism, secondary metabolism, cytochrome P450, transport activity and hormone metabolism may be responsible for the unique color and shape of lip. A group of MADS box transcription factors were also included. Clustering analyses of the 168 morphology-related genes showed that the expression profile of the peloric petal is much more similar to that of the lip (from wild-type or peloric mutant) than to that of the petal (Figure 5F), indicating the significance of this group of genes in the orchid lip development. Among these differentially expressed genes, MADS box transcription factors such as *PaSOC1-3*, *PaAGL6-2* and *PaAP3-3* may play critical roles in the lip formation or the maintenance of homeostasis. This is suggested by the Principal Component Analysis (PCA) with high loading scores and provides a potential transcriptional activation network for future studies. 

Thirty of the 168 genes that were postulated to have topological effects (Figure 5C), genes for several enzymes such as phenylalanine ammonia-lyase (PATC156804) and tyrosine decarboxylase (PATC138942 and PATC149447) that are involved in amino acid metabolism appeared to be expressed at higher levels in the lip than in the petal. In contrast, a putative zinc finger protein gene (PATC149247) was expressed at a higher level in the petal than in the lip. Genes for leucine-rich repeat transmembrane protein kinase (PATC139333) and anther-specific proline-rich protein (PATC147735) showed the same trend in their expression patterns. Two acyltransferase genes, *PATC134853* and *PATC196836*, showed opposite expression patterns, with PATC134853 having a higher expression levels in the petal and PATC196836 having a higher level in the lip. These genes seemed to maintain their spatial expression pattern regardless of the morphological changes in the peloric petal.

**Figure 6:**
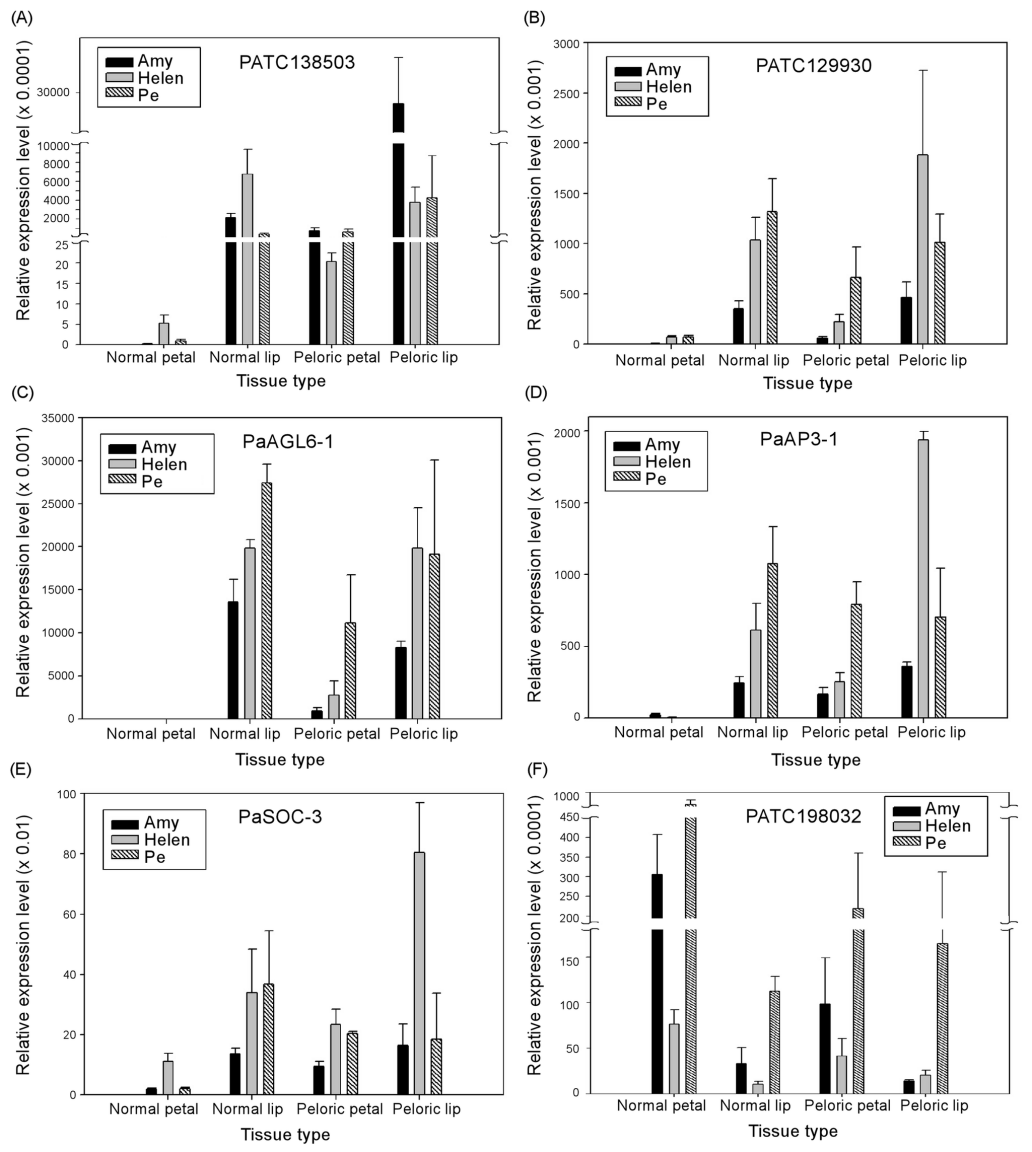
Quantitative PCR results to validate genes specifically expressed during morphological differentiation. Amy: P. Nobby’s Amy; Helen: Dtps I-Hsing Helen; Pe: *Phalaenopsis* *equestris*. Validated genes include, (A) *PaMTN3* (PATC138503); (B) *PaZF* (PATC129930); (C) *PaAGL6-1*; (D) *PaAP3-3*; (E) *PaSOC1-3*; (F) PATC198032. Primers used in the PCR reactions are listed in Table S3 in File S1.

Genes with petal/lip differentiation were chosen for validation with quantitative PCR (Figure 6). Many of the transcription factors such as *PaAGL6-1*, *PaAP3-3*, *PaZF* (zinc finger protein, PATC129930) and *PaSOC1-3* (PATC154491) that were expressed in the normal lip but not in the petal of the wild-type flower were found to be expressed in the peloric petal. On the contrary, PATC198032 (a MADS box gene) was expressed highly in the normal petal but not in the peloric petal and lip (Figure 6F). These results are consistent with our hypothesis that these transcription factors play essential roles in regulating floral identity. 

The factors that are involved in the determination of bilateral symmetry of orchid flowers have not been reported. Both *TCP* (characterized by a *CYCLOIDEA*-like domain, known as *CYC*) and MYB transcription factors have been suggested to be involved in determining the symmetric pattern of the *Antirrhinum* flower [56]. In *Gerbera hybrida*, a member of the Asteraceae, a TCP transcription factor (GhCYC2) was demonstrated to play an important role in the flower symmetry control [57]. We were unable to identify *CYCLOIDEA* (*CYC*) genes from the *Phalaenopsis* transcriptome. Whether there is an as yet unidentified orchid *CYC* homologous gene or specialized orchid transcription factor that regulates the bilateral symmetry of orchid flower remains to be clarified.

## Conclusions

Using a microarray, we conducted a large-scale investigation of the expression profiles of functional genes that encode transcription factors in *P. aphrodite*. Genes specifically expressed in the floral organs were analyzed for their potential roles in floral organ identity. Several major conclusions can be drawn from our studies. First, *PaAGL6-1*, which was expressed specifically in the orchid lip, may play an essential role in lip formation. Second, extending the expression of class B MADS box genes, *PaAP3-1* and *PaPI-1*, in the sepal may be significant for tepal formation. Third, clusters of genes involved in the morphological differentiation of the lip were further confirmed by a comparison of expression profiles between flowers of wild-type and a peloric mutant. Taken together, our results led to the proposal of a modified ABCDE model for orchids that accounts for mechanisms of flower morphorgenesis unique to orchids, and suggest that new classes of transcription factors have evolved to control the formation of floral organs in orchids. 

## Supporting Information

File S1 **Supporting figures and tables**. Figure S1. Performance check of orchid specialized microarray with scatter plot. Table S1. Top 20 genes differentially expressed in specific tissues. Figure S2. Quantitative PCR validation of genes differentially expressed in specific tissues. Table S2. Genes from the microarray clustering assay that were validated by quantitative PCR.Table S3. List of primers used for quantitative PCR analysis. Figure S3. Phylogenetic analysis of MADS box genes and AP2 genes. Table S4. MADS box genes and AP2 genes in phylogenetic analysis. Figure S4. Subcellular localization of MADS box genes according to particle bombardment. Figure S5. Peloric flowers of orchids.
(PDF)
