# Supplementary material for: A Modified ABCDE Model of Flowering in Orchids Based on Gene Expression Profiling Studies of the Moth Orchid Phalaenopsis aphrodite
Source: PLoS One. 2013 Nov 12;8(11):e80462. doi: 10.1371/journal.pone.0080462 (PMC3827201; doi:10.1371/journal.pone.0080462)
Supplement: File S1 — Supporting figures and tables. Figure S1. Performance check of orchid specialized microarray with scatter plot. Table S1. Top 20 genes differentially expressed in specific tissues. Figure S2. Quantitative PCR validation of genes differentially expressed in specific tissues. Table S2. Genes from the microarray clustering assay that were validated by quantitative PCR.Table S3. List of primers used for quantitative PCR analysis. Figure S3. Phylogenetic analysis of MADS box genes and AP2 genes. Table S4. MADS box genes and AP2 genes in phylogenetic analysis. Figure S4. Subcellular localization of MADS box genes according to particle bombardment. Figure S5. Peloric flowers of orchids. (PDF) [file pone.0080462.s001.pdf]

Supplementary Data

Features and quality assessment of *Phalaenopsis* specialized microarray

To examine genome-wide gene expression profiling of orchids, a customized microarray chip based on sequence information in the *Orchidstra* database [1] (http://orchidstra.abrc.sinica.edu.tw) was designed. The *Orchidstra* database contains 42,661 transcript contigs of *Phalaenopsis aphrodite* generated by *de novo* assembly procedure of reads generated from next generation sequencing (NGS) technologies such as Illumia Solexa and Roche 454 platforms. These transcript contigs were submitted to Agilent e-array (Agilent, CA) for microarray probe selection. Probe design of 39,431 transcript contigs was successful after software screening. Probes for the detection of common orchid viruses, Cymbidium mosaic virus (CymMV) and Odontoglossum ringspot virus (ORSV), were included to array design.

We have performed a series of quality test on the tailor made orchid array. Technical repeats showed reproducibility with correlation coefficients higher than 99.9% (after percentile shift normalization at 75%) between repeats (Supplementary Figure S1A). On average, 64-72% whole probe-sets on the array could be considered as detectable under Agilent scanner criteria or raw intensity more than 50. The detection rate was reasonable for basal expression since we have compared various orchid tissues including leaf, root, flower and germinating seeds under normal culture environment. The array performance between the diploid *Phalaenopsis aphrodite*, a Taiwan native species, and the tetraploid Sogo Yukidian 'V3', a popular commercial hybrid of *Phalaenopsis*, was satisfactory with correlation coefficients higher than 94% when same tissues were compared (Supplementary Figure S1B). The application of this array is therefore not limited to the rather scarce source of native *Phalaenopsis* orchid and can be extended to the more popular commercial hybrid.

(A)

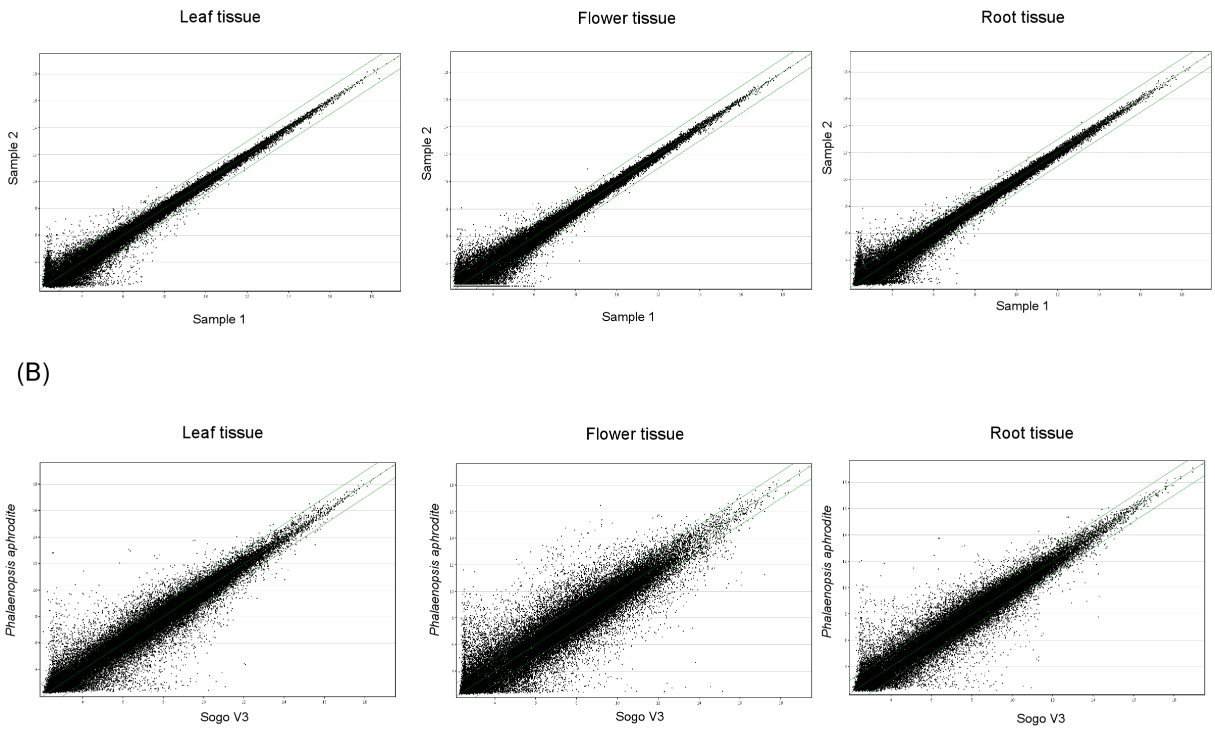

Figure S1. Performance check of orchid specialized microarray with scatter plot. (A) Overall comparative profiling between tissue samples of *Phalaenopsis aphrodite*. (B) Overall comparative profiling between *Phalaenopsis aphrodite* and the commercial hybrid Sogo Yukidian ‘V3’.

Differentially expressed gene list

Table S1. Top 20 genes differentially expressed in specific tissue. Expression level was determined after normalization of microarray intensities and SPM value (Specificity Measure) was calculated accordingly. Maximum value of SPM is 1. Top 20 genes with the highest SPM value are listed.

Probe Name	Description	SPM value
**Flower**		
PATC150065 (PaMYB-4)	Similar to ACB59077,Myb-like transcription factor EOBII [Petunia x hybrida]	1
PATC138068	Similar to XP_002520171, ER glycerol-phosphate acyltransferase [Ricinus communis]	1
PATC144452	Putative protein to AAO49418, MYB9 [Dendrobium sp. XMW-2002-9]	0.999999
PATC134411	Weak similar to XP_002889772,DNA polymerase delta subunit 4 family [Arabidopsis lyrata subsp.lyrata]	0.999999
PATC156712	Similar to CAD87008, MYB8 protein [Gerbera hybrid cv. 'Terra Regina']	0.999997
PATC118359	Weak similar to BAF98468, cytochrome P450 [Coptis japonica var. dissecta]	0.999996
PATC154654	Homologue to ACC59773, flavonoid 3',5' hydroxylase-like protein [Vanda coerulea]	0.999988
PATC138772 (PaAGL6-2)	Homologue to ADI58464, AGL6 [Cymbidium goeringii]	0.999987
PATC136147	Similar to CBI16443, unnamed protein product [Vitis vinifera]	0.999987
PATC112668	Similar to EEC77471, hypothetical protein OsI_16293 [Oryza sativa Indica Group]	0.999987
PATC140326	Similar to XP_002512728, "ATP-binding cassette transporter, putative [Ricinus communis]"	0.999986
PATC145620	Similar to XP_002516348, Sorting nexin-4, putative [Ricinus communis]	0.999985
PATC152417	Similar to XP_002463916, hypothetical protein SORBIDRAFT_01g008880 [Sorghum bicolor]	0.999985
PATC009198	Putative protein to CAA71513, putative cytochrome P450 [Glycine max]	0.999984
PATC209271	Weak similar to XP_002518849, 3-oxoacyl-[acyl-carrier-protein]	0.999984
PATC124681	Weak similar to BAD16430, putative AP2 domain-containing protein [Oryza sativa JaponicaGroup]	0.999983
PATC138420	Similar to XP_002521076, Xyloglucan endotransglucosylase/hydrolase protein 2 precursor, putative [Ricinus communis]	0.999981
PATC143361	Similar to EAY88298, hypothetical protein OsI_09755 [Oryza sativa Indica Group]	0.99998
PATC131581	Similar to XP_002885644, hydrolase, alpha/beta fold family protein Arabidopsis lyrata subsp. lyrata]	0.999691

PATC124610	Putative protein to BAD44483, hypothetical protein [Arabidopsis thaliana]	0.997148
**Lip**		
PATC138503 (PaMTN3)	Similar to NP_001149087, MTN3, [Zea mays]	1
PATC126569	Similar to ADG34844, putative phenylacetaldehyde synthase [Vanda hybrid cultivar]	0.999911
PATC034663	Putative protein to BAF96951, flavone synthase II [Iris x hollandica]	0.999234
PATC130766	Putative protein to EAZ04731, hypothetical protein OsI_26893 [Oryza sativa Indica Group]	0.998892
PATC138606	Similar to XP_002265207, 3-oxoacyl-[acyl-carrier-protein] synthase[Vitis vinifera]	0.998676
PATC209271	Weak similar to XP_002518849, 3-oxoacyl-[acyl-carrier-protein]	0.998214
PATC190376	Weak similar to CAA58732, PAR-1c [Nicotiana tabacum]	0.997444
PATC201263	Putative protein to ACP27626, beta-carotene hydroxylase [Oncidium Gower Ramsey]	0.996435
PATC147953	Similar to ABF93742, beta-carotene hydroxylase, putative, expressed [Oryza sativa(japonica cultivar-group)]	0.996286
PATC177749	Similar to NP_191000, WRI1 (WRINKLED 1); DNA binding / transcription factor [Arabidopsisthaliana]	0.995604
PATC067105	Similar to ABJ90468, beta-ketoacyl-ACP synthase I, [Jatropha curcas]	0.993919
PATC187105	Weak similar to ACG45585, cytochrome P450 CYP734A8 [Zea mays]	0.993342
PATC025229	Similar to BAF02551, putative brassinosteroid hydroxylase [Solanum lycopersicum]	0.993267
PATC132514	Similar to XP_002301652, acyl:coa ligase acetate-coa synthetase-like protein [Populus trichocarpa]	0.993036
PATC199654	Weak Similar to NP_200018, MATE efflux protein-related [Arabidopsis thaliana]	0.992537
PATC154379 (PaAGL6-1)	Homologue to ADJ67237, MADS box transcription factor 1 [Oncidium Gower Ramsey]	0.992168
PATC023454	Similar to XP_002301652, acyl:coa ligase acetate-coa synthetase-like protein [Populus trichocarpa]	0.991833
PATC030120	Weak Similar to NP_194643, MATE efflux protein-related [Arabidopsis thaliana]	0.991163
PATC166947	Weak Similar to XP_002307962, acyl:coa ligase acetate-coa synthetase-like protein [Populus trichocarpa]	0.9903
PATC164233	Similar to ACG32367, AP2/EREBP transcriptional factor WRI1 [Zea mays]	0.990028
**Pollinia**		
PATC024902	Putative protein to XP_002308395, AP2 domain-containing transcription factor [Populus trichocarpa]	1
PATC138224	Similar to XP_002325139, AP2 domain-containing transcription factor [Populus trichocarpa]	1
PATC056313	Putative protein to XP_003027790, ubiquitin-conjugating enzyme E2 [Schizophyllum commune H4-8]	1
PATC142684	Weak similar to XP_002278602, PREDICTED: hypothetical protein [Vitis vinifera]	1
PATC154622	Similar to ACG56678, tryptophan aminotransferase [Zea mays]	1

PATC138414	Putative protein to ACG33223, pollen-specific protein C13 precursor [Zea mays]	1
PATC125413	Similar to AAY86035, pyruvate kinase [Citrus sinensis]	1
PATC134337	Similar to ACF06586, aluminum-induced protein [Elaeis guineensis]	1
PATC154983	Putative protein to ACG33223, pollen-specific protein C13 precursor [Zea mays]	1
PATC155194	Putative protein to XP_002325139, AP2 domain-containing transcription factor [Populus trichocarpa]	1
PATC055518	Similar to NP_001063510, NP_001063510.1, Os09g0483500, [Oryza sativa Japonica Group] Calcium-binding EF-hand domain containing protein	1
PATC059937	Weak similar to AAW88315, expansin EXPA11, [Triticum aestivum]	1
PATC120711	Putative protein to XP_002884013, ubiquitin-conjugating enzyme 27 [Arabidopsis lyrata subsp. lyrata]	1
PATC138059	Weak similar to XP_002308395, AP2 domain-containing transcription factor [Populus trichocarpa]	1
PATC150528	Similar to XP_002532661, serine-threonine protein kinase, putative [Ricinus communis]	1
PATC194643	Similar to ABM54492, expansin 2 [Cunninghamia lanceolata]	1
PATC058317	Similar to AAL99224, ubiquitin-conjugating enzyme E2 [Gossypium thurberi]	1
PATC138131	Similar to CAQ58629, pyruvate kinase [Vitis vinifera]	1
PATC154132	Similar to BAG80542, glycosyltransferase [Lycium barbarum]	1
PATC191966	Putative protein to ABS32237, asymmetric leaves 2 [Carica papaya]	1
PATC133027	Similar to BAI52955, calcium-binding EF-hand protein [Citrullus lanatus subsp. vulgaris]	1
PATC118656	Similar to BAC81184, putative glucose transport protein STP1 [Oryza sativa Japonica Group]	1
PATC036239	Putative protein to XP_002519123, F-box and wd40 domain protein, putative [Ricinus communis]	1
PATC138345	Putative protein to XP_002325139, AP2 domain-containing transcription factor [Populus trichocarpa]	1
PATC137385	Similar to ABJ99596, pyruvate decarboxylase [Lycoris aurea]	1
**Column**		
PATC154343	Similar to XP_002529091, Polygalacturonase precursor, putative [Ricinus communis]	0.999999
PATC125905	Homologue to CAA56277, bibenzyl synthase [Phalaenopsis sp. 'pSPORT1']	0.999712
PATC130846	Similar to 1Z3Q, Chain A, Resolution Of The Structure Of The Allergenic AndAntifungal Banana Fruit Thaumatin-Like Protein At 1.7a	0.999641
PATC197430	Putative protein to XP_002265159, PREDICTED: hypothetical protein [Vitis vinifera]	0.998991
PATC138585 (PaAG-2)	Homologue to AAZ95250, AGAMOUS-like transcription factor [Dendrobium crumenatum]	0.998456
PATC038602	Similar to XP_002514682, UDP-glucuronosyltransferase, putative [Ricinus communis]	0.997282
PATC190622	Putative protein to XP_002513511, transferase, transferring glycosyl groups,putative [Ricinus communis]	0.997046

PATC165907		Similar to XP_002526116, UDP-glucuronosyltransferase, putative [Ricinus communis]	0.997042
PATC147520		Putative protein to BAD43783, putative bHLH transcription factor (bHLH037) [Arabidopsis thaliana]	0.996157
PATC036637		Similar to XP_002526116, UDP-glucuronosyltransferase, putative [Ricinus communis]	0.996157
PATC116847		Putative protein to AAA32914, polygalacturonase [Persea americana]	0.9954
PATC119249		Weak similar to AAL76415, MADS-box transcription factor [Phalaenopsis equestris]	0.995019
PATC165547		Similar to XP_002526116, UDP-glucuronosyltransferase, putative [Ricinus communis]	0.994654
PATC002540		Putative protein to BAC57273, putative polygalacturonase PG1 [Oryza sativa Japonica Group]	0.994233
PATC009554		Putative protein to XP_002526107, UDP-glucuronosyltransferase, putative [Ricinus communis]	0.993436
PATC000227		Putative protein to BAC10994, rhamnosyl transferase [Nierembergia sp. NB17]	0.993142
PATC061977		Putative protein to AAY86364, AGAMOUS-like protein [Dendrobium thyrsiflorum]	0.992483
PATC052530		Similar to XP_002520171, ER glycerol-phosphate acyltransferase [Ricinus communis]	0.992352
PATC140136		Similar to NP_001152613, MYB-CC type transfactor [Zea mays]	0.991949
PATC198687		Putative protein to NP_001064591, Os10g0413400 [Oryza sativa Japonica Group]	0.991353
PATC155109	(PaAG-3)	Homologue to AAL76415, MADS-box transcription factor [Phalaenopsis equestris]	0.99129
**Pedicel**			
PATC148981		Weak similar to AAQ11882, knotted 1 [Hordeum vulgare]	0.999996
PATC175072		Putative protein to ACI23247, COMPOUND INFLORESCENCE [Solanum lycopersicum]	0.99991
PATC022841		Homologue to ADI58462, flowering locus T [Cymbidium goeringii]	0.999904
PATC173815		Putative protein to ACL11801, WOX9-like protein [Phaseolus coccineus]	0.999818
PATC145039		Similar to XP_002510423, cytochrome P450, putative, [Ricinus communis]	0.999631
PATC145786		Similar to CAB88029, knotted1-like homeobox protein [Dendrobium grex Madame Thong-In]	0.999388
PATC127065		Similar to ABI74672, class I KNOX-like 1 protein [Elaeis guineensis]	0.999125
PATC150662		Similar to ABU88887, S-adenosyl-L-methionine:salicylic acid carboxyl methyltransferase [Chimonanthus praecox]	0.99827
PATC155095		Similar to CAD70566, carboxyl methyltransferase [Crocus sativus]	0.997303
PATC150515		Putative protein to XP_002272432, PREDICTED: hypothetical protein [Vitis vinifera]	0.997057
PATC166128		Putative protein to BAH83538, DL related protein [Triticum aestivum]	0.996476
PATC144516		Similar to BAI49293, homeodomain leucine zipper protein class 1 [Hordeum vulgare subsp. vulgare]	0.994982
PATC074709		Putative protein to AAW83045, CRABS CLAW [Capparis flexuosa]	0.99446

PATC147749	Similar to BAF93480, class-I knotted1-like homeobox protein IBKN3 [Ipomoea batatas]	0.992282
PATC163884	Weak similar to XP_002517072, conserved hypothetical protein [Ricinus communis]	0.991868
PATC069479	Similar to ABF70015, zinc finger (Dof type) family protein [Musa acuminata]	0.989296
PATC069726	Putative protein to XP_002284464, PREDICTED: similar to bHLH transcription factor Upa20 [Vitisvinifera]	0.988977
PATC122106	Putative protein to XP_002526340, LOB domain-containing protein, putative [Ricinus communis]	0.987073
PATC148697	Similar to ACN21632, putative basic helix-loop-helix protein BHLH7 [Lotus japonicus]	0.986846
PATC202120 (PaAG-4)	Putative protein to AAZ95251, AGAMOUS-like transcription factor [Dendrobium crumenatum]	0.985674

Quantitative PCR for validation of tissue specific expression pattern

Method

All the RNA samples were treated with DNase treatment by TURBO DNA-free kit (Ambion, TX, USA) and quantified by RNA Bioanalyzer (Agilent, CA, USA). cDNA was synthesized from 1 μg of total RNA with M-MLV Reverse transcrptase kit (Invitrogen, CA, USA) and poly T primer. All primers used were designed by Primer Express version 3.0 (Applied Biosystems, CA, USA). A total of 20 μl real-time PCR reaction contained primers, cDNA and 10 μl 2X SYBR Green PCR master mix (Applied Biosystems, CA, USA). Real-time PCR was performed in the ABI Prism 7300 Sequence Detection System (Applied Biosystems, CA, USA) with programs recommended by the manufacturer (2 min at 50 ^{o}C, 10 min at 95 ^{o}C and 40 cycles of 95 ^{o}C for 15 sec and 60 ^{o}C for 1 min). Each sample was performed with real-time PCR for three independent biological replicates. The comparative C_T method (cycle of threshold) was used to determine the relative level of gene expression, with the expression value of ubiquitin (PATC150470) or actin (PATC135993) used as internal controls. Relative expression level is determined by delta Ct of target gene normalized to the internal control. Genes for the validation are listed in Supplementary Table S2 and their primers were listed in Table S3.

(A) Whole flower specific

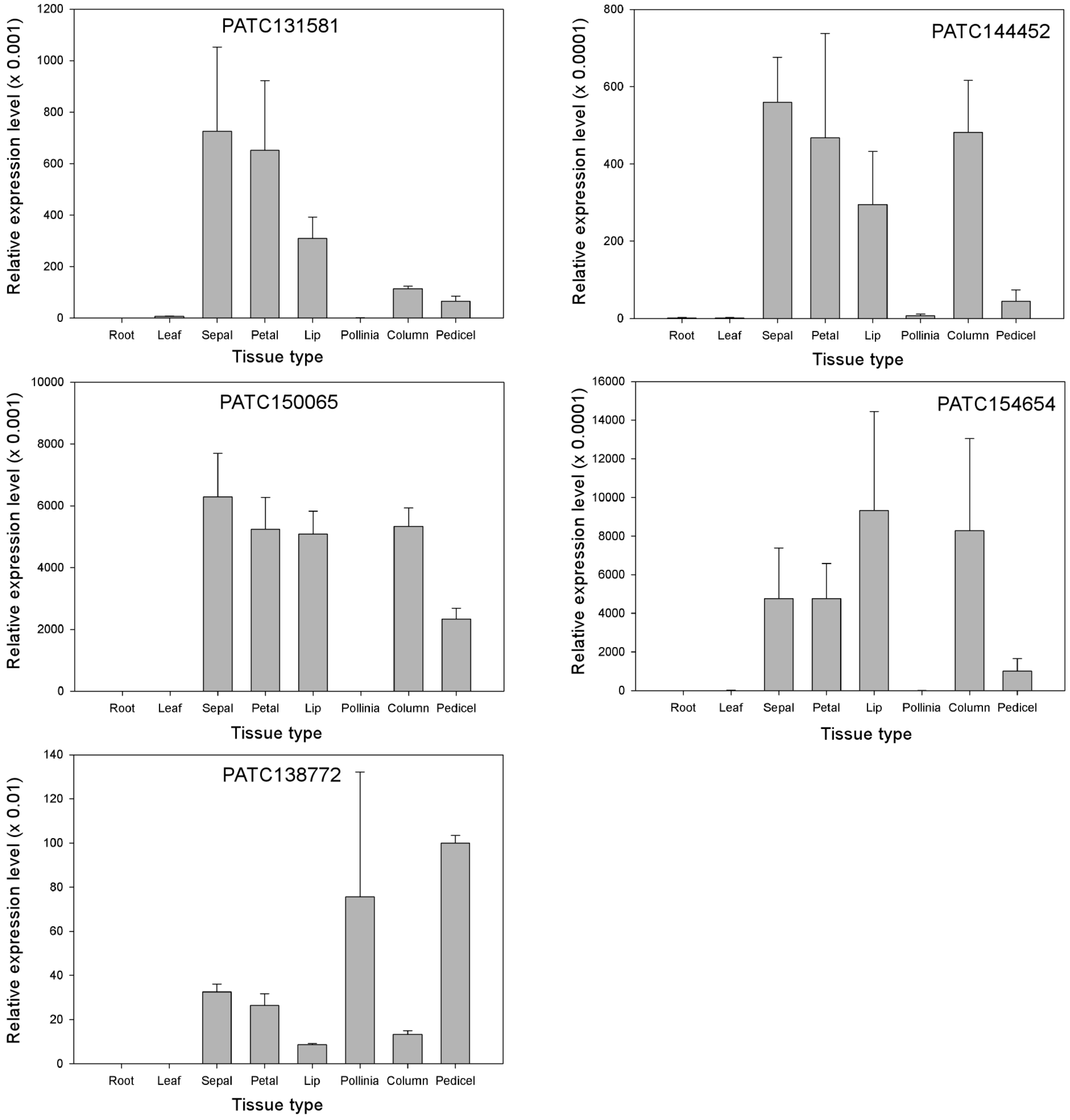

(B) Lip specific

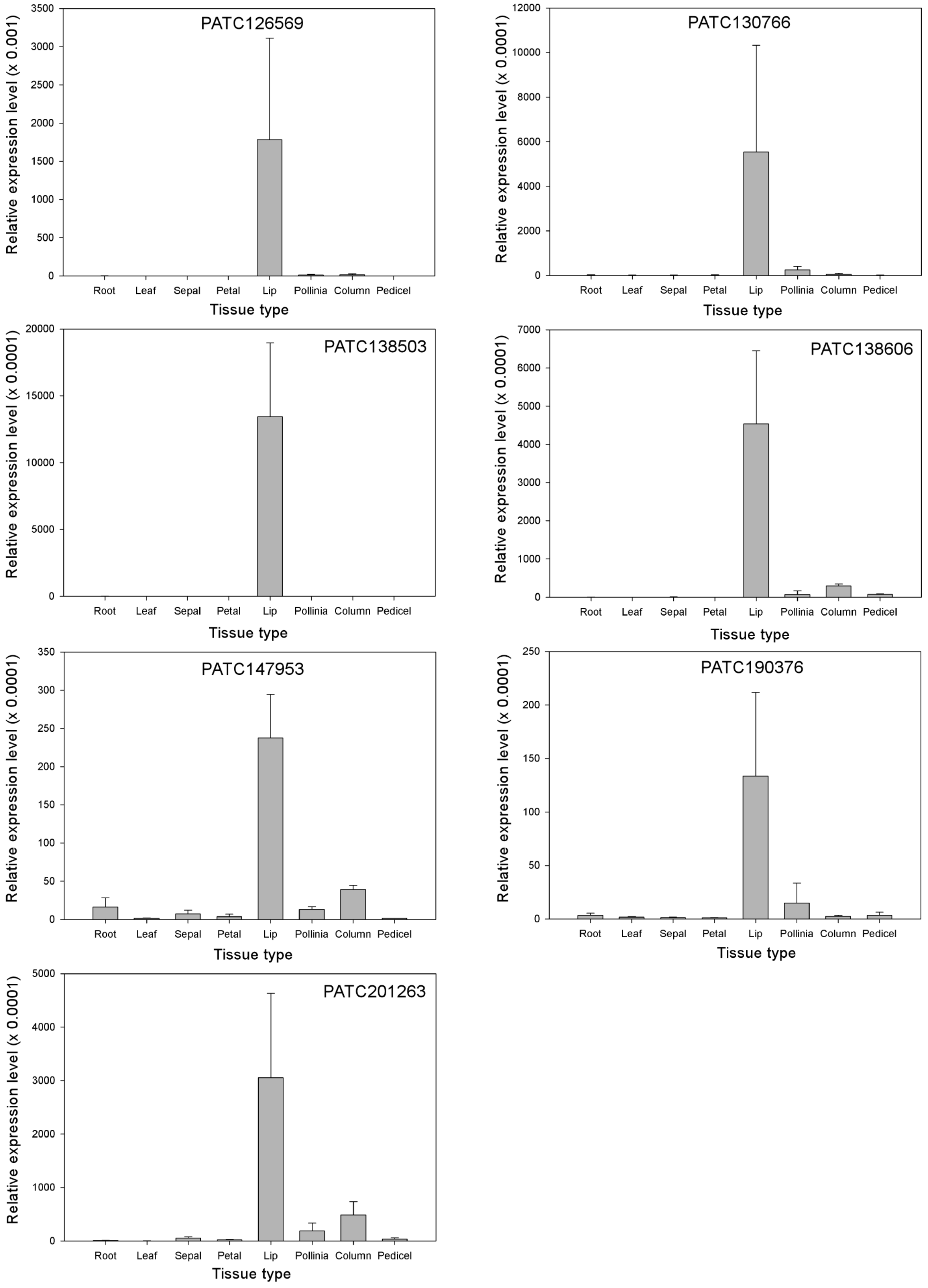

(C) Pollinia specific

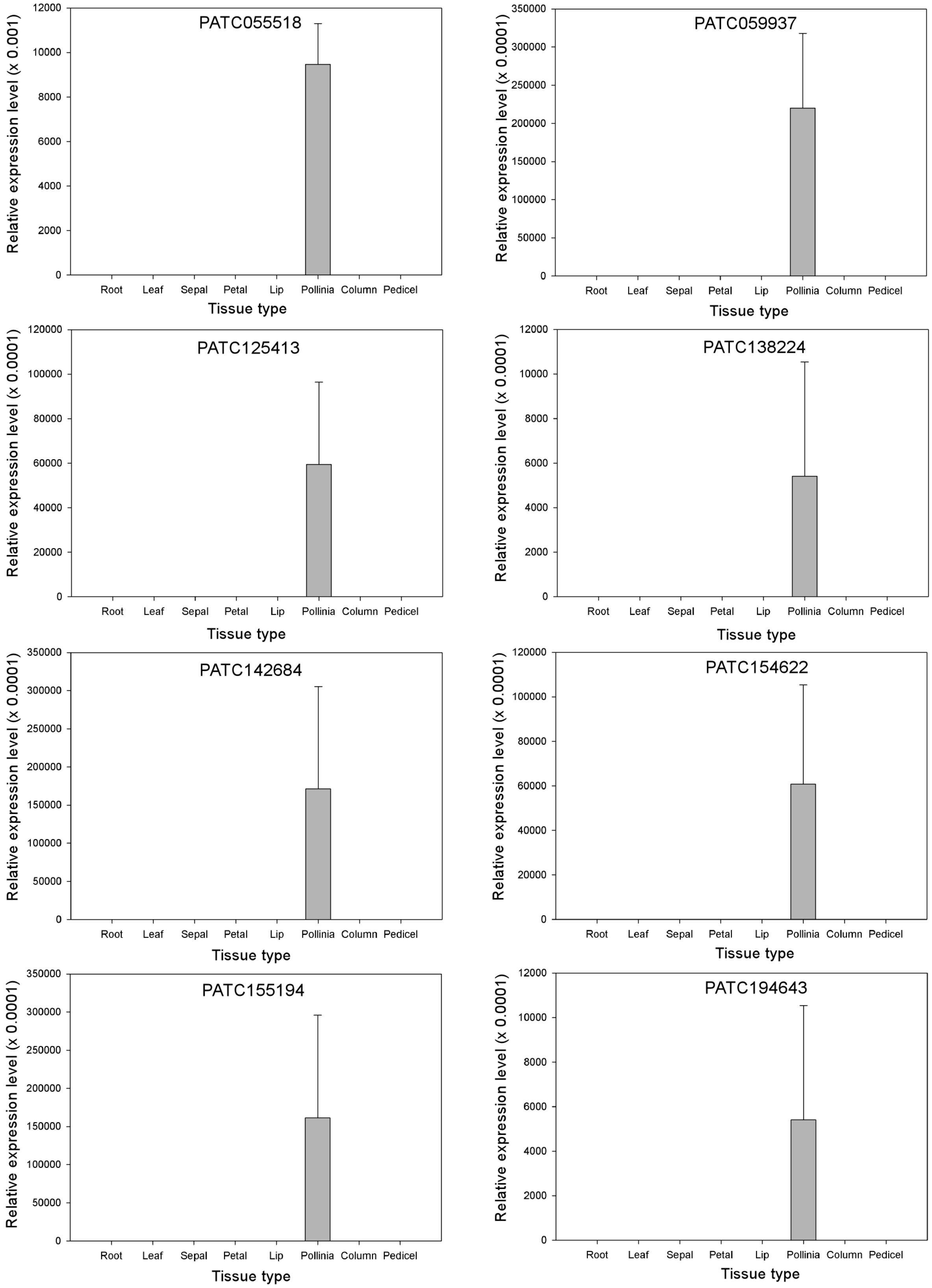

(D) Column specific

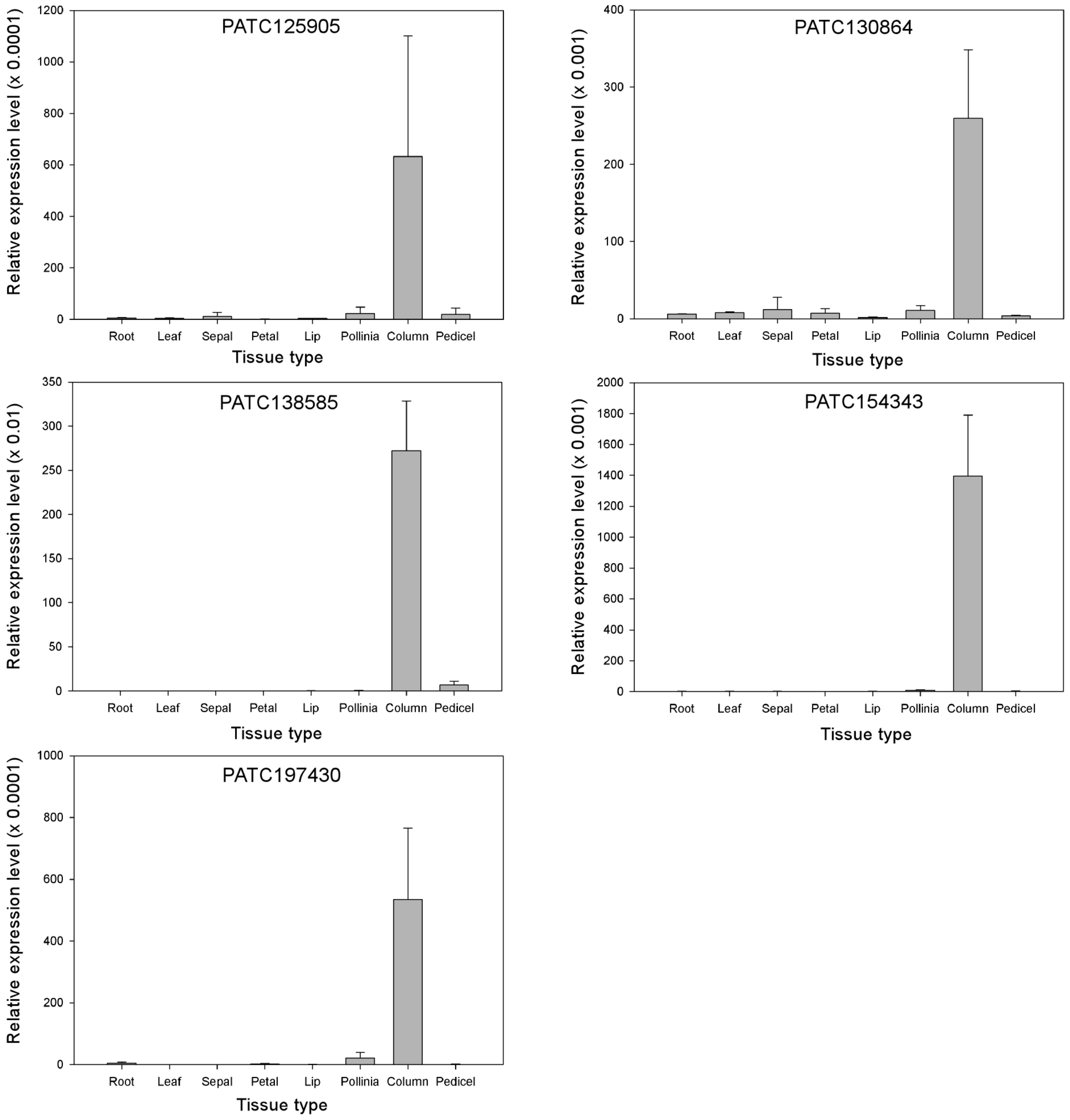

(E) Pedicel specific

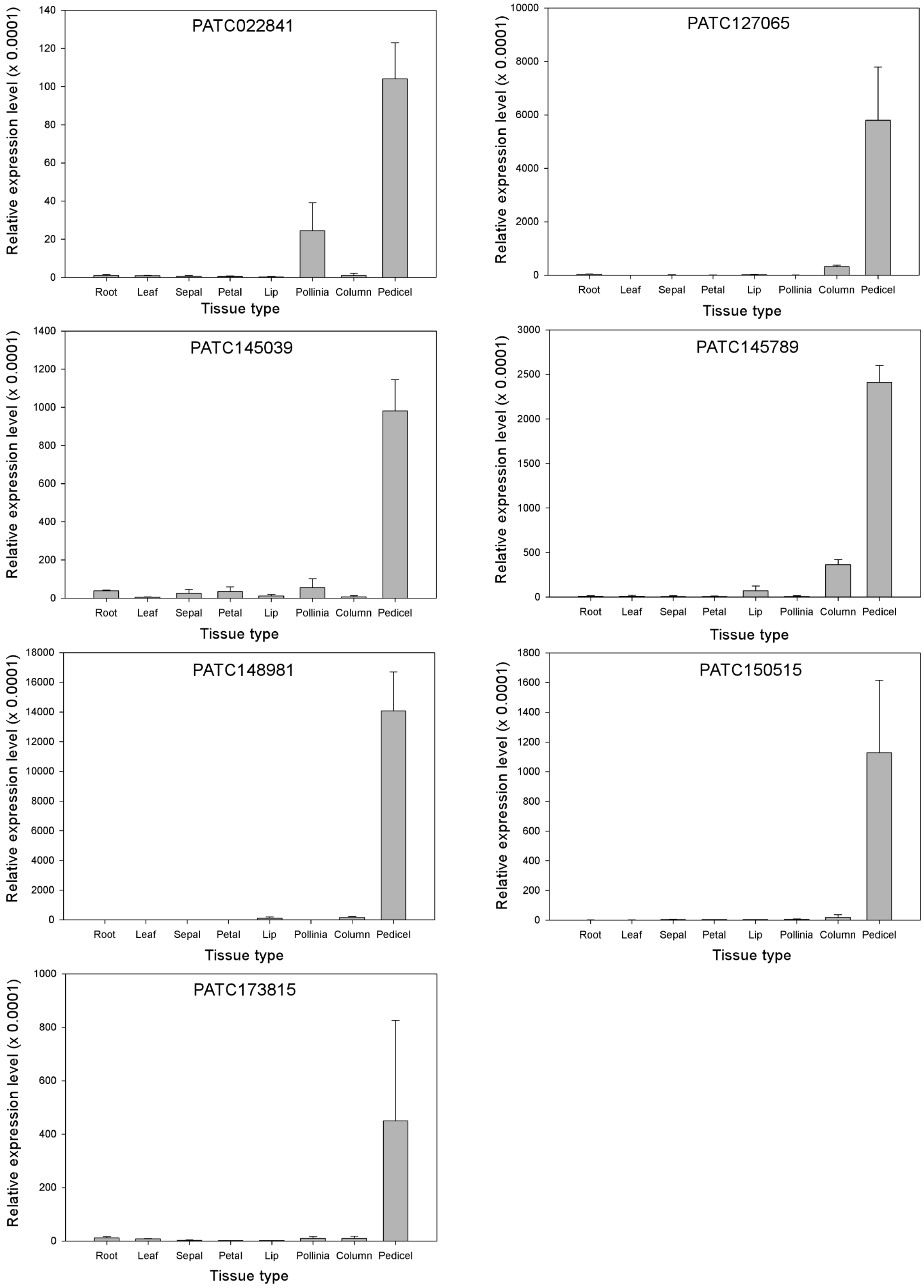

Figure S2. Quantitative PCR validation of genes differentially expressed in specific tissues. Relative expression level was normalized to a house-keeping gene, Ubiquitin (PATC150470),

with the determination of PCR cycle of threshold. Experiments with triple biological repeats were performed. Standard deviation was shown on figure. (A) Flower specific expression pattern. (B) Lip specific expression pattern. (C) Pollinia specific expression. (D) Column specific pattern. (E) Pedicel specific expression. Primers for the assay were listed in Supplementary Table S3.

Table S2. Genes from the microarray clustering assay that were validated by quantitative PCR. Ubiquitin gene (PATC150470) and actin (PATC135993) were used as internal controls for normalization. Gene-specific primers for validation were listed in Supplementary Table S3.

Gene id	Gene description
**Flower specific (Figure S2A)**	
PATC131581	Similar to XP_002885644, hydrolase, alpha/beta fold family protein Arabidopsis lyrata subsp. lyrata]
PATC150065 (PaMYB-4)	Similarto ACB59077, Myb-like transcription factor EOBII [Petunia x hybrida]
PATC154654	Homologue to ACC59773, flavonoid 3',5' hydroxylase-like protein [Vanda coerulea]
PATC144452	Putative protein to AAO49418, MYB9 [Dendrobium sp. XMW-2002-9]
PATC138772 (PaAGL6-2)	Homologue to ADI58464, AGL6 [Cymbidium goeringii]
**Lip specific (Figure S2B)**	
PATC138503 (PaMTN3)	Similar to ref|NP_001149087.1|MTN3 [Zea mays] (Sugar efflux transporter for intercellular exchange)
PATC126569	Similar to ADG34844, putative phenylacetaldehyde synthase [Vanda hybrid cultivar] (PAAS –catalyzes the formation of PHA (floral scent)
PATC130766	Putative protein to EAZ04731, hypothetical protein OsI_26893 [Oryza sativa Indica Group]
PATC138606	Similar to XP_002265207, 3-oxoacyl-[acyl-carrier-protein] synthase [Vitis vinifera]
PATC190376	Weak similar to emb|CAA58732.1| PAR-1c [Nicotiana tabacum]
PATC201263	Putative protein to ACP27626, beta-carotene hydroxylase [Oncidium Gower Ramsey]
PATC147953	Similar to >dbj|BAH10591.1| beta-ring hydroxylase [Lilium hybrid division I]
PATC177749	Similar to NP_191000, WRI1 (WRINKLED 1); DNA binding / transcription factor [Arabidopsisthaliana]
**Pollinia specific (Figure S2C)**	
PATC199170	Similar to ABM54492, expansin 2 [Cunninghamia lanceolata]
PATC138224	Similar to XP_002325139, AP2 domain-containing transcription factor Populus trichocarpa]
PATC142684	Putative protein to >ref|XP_002522563.1|Heterogeneous nuclear ribonucleoprotein A1, putative [Ricinus communis]
PATC154622	Similar to >gb|ACG56678.1| tryptophan aminotransferase Zea mays]
PATC125413	Similar to AAY86035, pyruvate kinase [Citrus sinensis]
PATC155194	Putative protein to >ref|XP_002325139.1| AP2 domain-containing transcription factor Populus trichocarpa]
PATC059937	Weak similar to >gb|AAW88315.1| expansin EXPA11 [Triticum aestivum]
PATC055518	Similar to NP_001063510, Os09g0483500 [Oryza sativa Japonica Group]

Column specific (Figure S2D)

PATC154343 Similar toXP_002529091, Polygalacturonase precursor, putative [Ricinus communis]

PATC125905 Homologueto CAA56277, bibenzyl synthase [Phalaenopsis sp. 'pSPORT1']

PATC130846 Similar to 1Z3Q, Chain A, Resolution Of The Structure Of The Allergenic AndAntifungal Banana Fruit Thaumatin-Like Protein At 1.7a

PATC197430 Putative protein to XP_002265159, PREDICTED: hypothetical protein [Vitis vinifera]

PATC138585 (PaAG-2) Homologue to AAZ95250, AGAMOUS-like transcription factor [Dendrobium crumenatum]

Pedicel specific (Figure S2E)

PATC148981 Weak similar to AAQ11882, knotted 1 [Hordeum vulgare]

PATC022841 Homologue to ADI58462, flowering locus T [Cymbidium goeringii]

PATC173815 Putative protein to >gb|ACL11801.1| WOX9-like protein Phaseolus coccineus]

PATC145039 Similar to XP_002510423, cytochrome P450, putative Ricinus communis]

PATC145786 Similar to CAB88029, knotted1-like homeobox protein [Dendrobium grex Madame Thong-In]

PATC127065 Similar to ABI74672, class I KNOX-like 1 protein [Elaeis guineensis]

PATC150515 Putative protein to XP_002272432, PREDICTED: hypothetical protein [Vitis vinifera]

Transcription factors (Figure 5C, 5D)

PATC135043 (PabHLH-1) Similar to NP_001031255, basic helix-loop-helix (bHLH) family protein [Arabidopsis thaliana]

PATC150065 (PaMYB-4) Similar to ADG58063, transcription factor [Lycoris longituba]

PATC134262 (PabZIP-2) Similar to NP_172097, "bZIP transcription factor, putative (bZIP69) [Arabidopsis thaliana]"

PATC133172 Similar to ADJ67440, ethylene response factor 11 [Actinidia deliciosa]

PATC136849 (PabZIP-3) Weak similar to AAK92213, bZIP transcription factor BZI-2 [Nicotiana tabacum]

PATC140443 (PabHLH-3) Similar to ABR23669, Myc2 bHLH protein [Vitis vinifera]

PATC152106 (PaMYB-2) Similar to XP_002528315, r2r3-myb transcription factor, putative [Ricinus communis]

PATC138345 (PaAP2-7) Putative protein to XP_002325139, AP2 domain-containing transcription factor [Populus trichocarpa]

PATC138298 (PaNAC-3) Similar to ADG57969, transcription factor [Lycoris longituba]

MADS box genes (Figure 5A, 5B)

PATC154379 (PaAGL6-1) Homologue to ADJ67237, MADS box transcription factor 1 [Oncidium Gower Ramsey]

PATC138772 (PaAGL6-2) Homologue to ADI58464, AGL6 [Cymbidium goeringii]

PATC155109 (PaAG-3) Homologue to AAL76415, MADS-box transcription factor [Phalaenopsis equestris]

PATC138798 (PaMδS-1)	Similar to NP_001148603, MADS-box protein AGL66 [Zea mays]
PATC138540 (PaSEP-1)	Homologue to ADJ67238, MADS box transcription factor 6 [Oncidium Gower Ramsey]
PATC154853 (PaAP3-4)	Homologue to AAR26629, MADS-box transcription factor [Asparagus officinalis]
PATC138350 (PaAP3-3)	Homologue to AAR26626, MADS box transcription factor [Phalaenopsis equestris]
PATC152852 (PaPI-1)	Homologue to AAV28175, MADS box PI-like protein 9 [Phalaenopsis hybrid cultivar]
**Morphological genes (Figure 6)**	
PATC154491 (PaSOC1-3)	Similar to ACV88635, SUPRESSOR OF OVEREXPRESSION OF CONSTANS1 [Magnolia virginiana]
PATC198032	Weak similar to ADJ67237, MADS box transcription factor 1 [Oncidium Gower Ramsey]
PATC129930 (PaZF)	Similar to AAO64176, putative zinc finger protein [Arabidopsis thaliana]
PATC138503 (PaMTN3)	Similar to NP_001149087, MTN3, [Zea mays]
**Internal control**	
PATC150470	Homologue to NP_001148325, ubiquitin-like protein SMT3 [Zea mays]
PATC135993	Similar to XP_002317939, actin related protein [Populus trichocarpa]

Table S3. List of primers used for quantitative PCR analysis.

Gene id	Forward primer (5' to 3')	Reversed primer (5' to 3')
**Flower specific (Figure S2A)**		
PATC131581	TCAAGCATGGGCTGAAGAATT	GCAAGGGAATGTGGTCGTTT
PATC150065 (PaMYB-4)	GCACCTGCACGCTTAAATTG	GAAGAGTCTGGACGGCGAAA
PATC154654	AATAAACCAATCATAAGCGATCAGAA	CCGACCGTTGGAAACTTTCA
PATC144452	CATCTTCACGATAGGTGCCATCT	TTGCAAACTAAAAGCAAAGCTCAA
PATC138772 (PaAGL6-2)	TTGTTCCTCCAGAAACTGTAATTCC	GCATCCATCCAAGCATAAAATTATT
**Lip specific (Figure S2B)**		
PATC138503 (PaMTN3)	TGGTGCGGCTCAAATTATACTTT	CAAGAGCCGATTCAGAAACCAT
PATC126569	CGCAGTGGGTCGTAGGTTTC	CCACACCAAATCGTCTGATGA
PATC130766	CCCCATCCGGTTTTATCAAA	CATCGCCGCTGGTATCGT
PATC138606	GGCGGTCAGATTCGAAATTTC	GCCTGCGTTCATTCTTTGC
PATC190376	CCGTTGCGTGCTTGAGAAG	CTTACGGCAGGAGTACTCATTGAA
PATC201263	GCTGAGCGGAGGTTGTATTTG	GCCGTAAATATGAATCCAGAGCTT
PATC147953	CCGTTTGAGGTCAACGATGTT	GGAGAGCAATGGCAGGGATA
**Pollinia specific (Figure S2C)**		
PATC199170	TAGCGCAAGGCTACGGCTTA	CAGCCCATCGTCGAACAGT
PATC138224	TCTCATTTCTCCGTCCAAATCC	CATCACAATTATCTCATTTCTCAAAGG
PATC142684	CCAACCGTGAGCCTCAAAA	TTTGGTTCCTTGGACTTGCAA
PATC154622	GGCTGGGCGGTAGTGAAA	CTCGACGAACTTCACCATTCTTC
PATC125413	CGATCTTGTCGGTGGTGGTT	TGACCGACCATTCGAATTTCT
PATC155194	GTTCGTGGTGTTACAAGCAGATG	TCTTGACGTGCTCTCCTCCAT
PATC059937	GCGAGGAGGAATGAGGTTCA	CCACATTCCACAACAGTATCATGTT

PATC055518	GCGAAGGAGCTTACGAACATTC	CCGATTGCCGTCAATGC
**Column specific (Figure S2D)**		
PATC154343	CGACAAAGGTGGCAGTGAAA	CGATTCCACTGCACGGATT
PATC125905	AGCGGCTAAGGCTTCATTCA	GGTGGGAAGTTTTTGTGATTTTG
PATC130846	TGTCCGGACGTACACTTATGTCA	GCTGAGAAAAGCATTTAAGACATCCT
PATC197430	CAATGCTTGTGGAGAAGGTGATAG	TGAAGCTTATCCATCCATAAATGG
PATC138585 (PaAG-2)	CTGATGGGTGAGGCACTTAGC	TCTCCAGTCTTGTTTCAAGTTGCT
**Pedicel specific (Figure S2E)**		
PATC148981	GAACGCTGCTGCTCTTTATATGG	AGGTCCGAATCGATAATTTCCA
PATC022841	TTCGGCAGTGAAATAGTGTGCTA	ACAAAGCGGTGTATGCCTAGACT
PATC173815	CATGACATCAACACCTCTTCTCTCTT	CGGATCCTGAAGCATAGGAGTT
PATC145039	ACAGGAACCCAAGCATAGCAA	TTGGGCAGCCACTCCTTAAC
PATC145786	TTTGATTGGTGGACTGCTCACTA	CAGCGCAATTTTGTCTGATTCT
PATC127065	CAGTTTAAGTCGCTCTCGCTTTC	TCAAGCTCACCACCCACTTG
PATC150515	TTCAAAGACTGGCCACAAACAT	TCTTCCCCCAACTCATTGTTG
**Transcription factors (Figure 5C, 5D)**		
PATC138345 (PaAP2-7)	CCCTCGACAATGTCTGGATTAGA	GCCTCCCTAGGCAAAGTCAA
PATC133172 (PaAP2-5)	CCGGCGATCTCAGCATCT	TGCCTCGCGGATCAGTATC
PATC135043 (PabHLH-1)	TGGCGTGCCTCGTGATG	GATAAGGCAGTGAACGAAAGGTCTT
PATC140443 (PabHLH-3)	TTATCGTGGGAGGTTCAGATCA	GAATTGTAGCCGGCGTTCA
PATC134262 (PabZIP-2)	TTAGTTGCTGGAGCGATGCA	ACATCCTTGATAAAATTGCCCTTAA
PATC136849 (PabZIP-3)	CATGTTTTTTAGTGTTCAGTTGTGAGTT	CGGCCTTCGCAACCTAATAA
PATC152106 (PaMYB-2)	GCGGTCCAGCCCTGACT	CAAATCAGCCCTCCTACACTGTT
PATC150065 (PaMYB-4)	GCACCTGCACGCTTAAATTG	GAAGAGTCTGGACGGCGAAA
PATC138298 (PaNAC-3)	CTATCACTCTTCGGCAGACCAA	ACTGCATGCTGGCTTCATCTAC
**MADS box genes (Figure 5A, 5B)**		

PATC154853 (PaAP3-4)	CCACCGATACCAAGAGTGTATATGA	GCTCGCTCCACAAATTTATGC
PATC152852 (PaPI-1)	AAAGAGCTTATTCCGATTGAGGAA	TGTTTATCCCGAACGCTAGTGA
PATC138540 (PaSEP-1)	CCGATTTATCGATTGCACCAA	CCAACCAGGTGGCATGTAATT
PATC154379 (PaAGL6-1)	GAGGTACAATCAGTATGCTCCTTCTG	CCAGGCATGAAAGTGTTTCCA
PATC138798 (PaMδS-1)	CAGATGCCTTGGCTCGTGTA	TTGTAGGCTTGGAGCAGATCGT
PATC155109 (PaAG-3)	CCACAAAACTGCGTCAGCAA	GCATCACCCAGCAAATTCCT
PATC138772 (PaAGL6-2)	TTGTTCCTCCAGAAACTGTAATTCC	GCATCCATCCAAGCATAAAATTATT
PATC138350 (PaAP3-3)	CCAAGAAACACACCGGAACTTAA	CCATAAACTGGGTGGTCCTCAA
PATC240636 (PaAP3-1)	GGGAAACTTATCGCGCTCTAATAC	CGTTCACCATTGGAATCGAATT
**Morphological genes (Figure 6)**		
PATC154491 (PaSOC1-3)	AGGAGATGGTTCCCCAATGTG	CGGGCCATCCTACATACAGTTC
PATC198032	AGACAATCGGACGATATCTACGGTGC	CTGGATCCATTGCTTGAGGCG
PATC129930 (PaZF)	TCTTAAGGATCATGTCAAGGCATTT	CAAAACAATGCACCCCACAAG
PATC138503 (PaMTN3)	TGGTGCGGCTCAAATTATACTTT	CAAGAGCCGATTCAGAAACCAT
**Internal Control**		
PATC135993 (Actin)	CTAGCGGAAACGCGACAGA	CCAAGGGAAGCCAAAATGC
PATC150470 (Ubiquitin)	GATCGCCAGTCGGTTGATTT	GAAGCCTACGGCCATCGA

Phylogeny analysis of MADS box gene and AP2 transcription factors

(A)

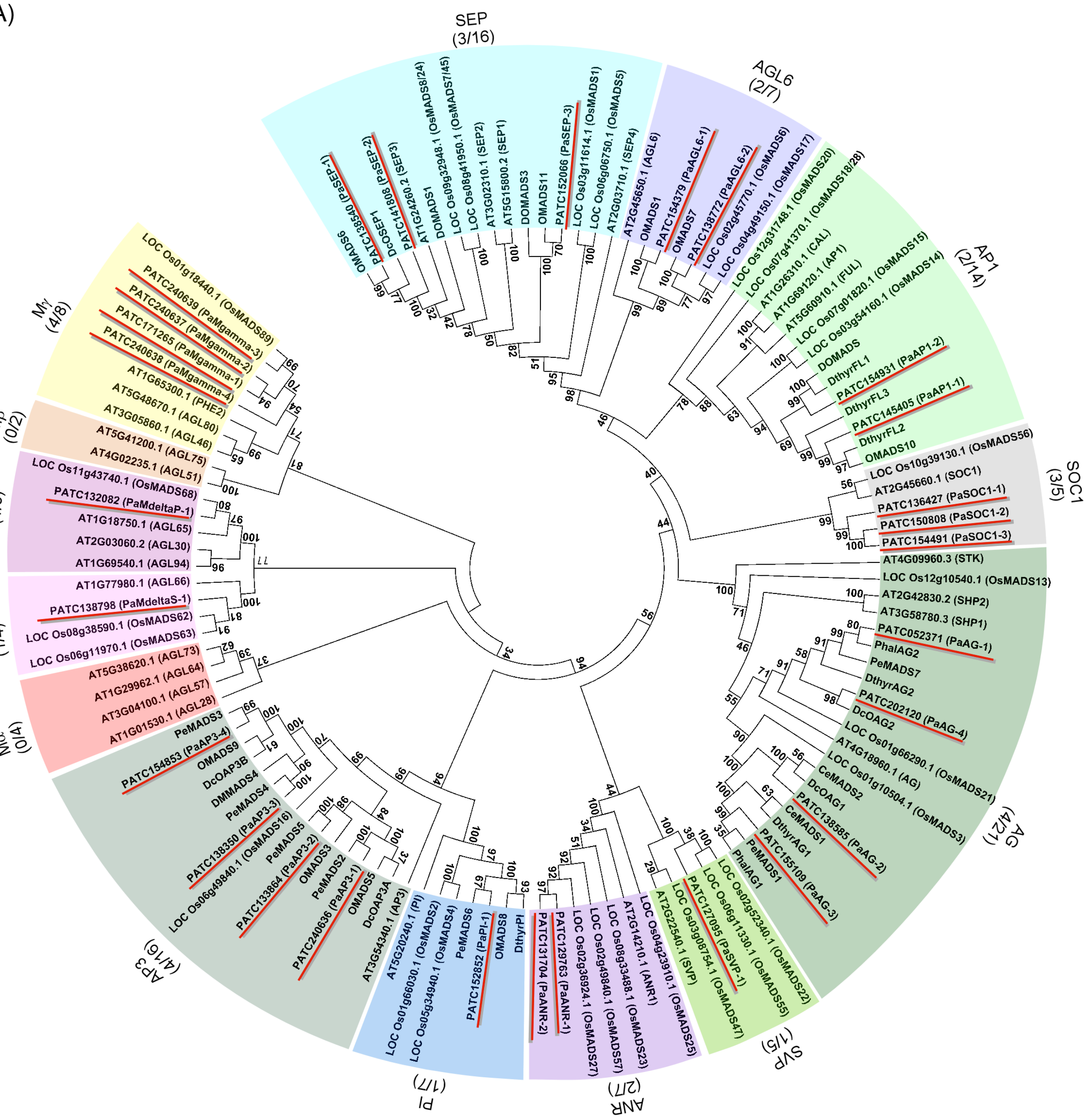

(B)

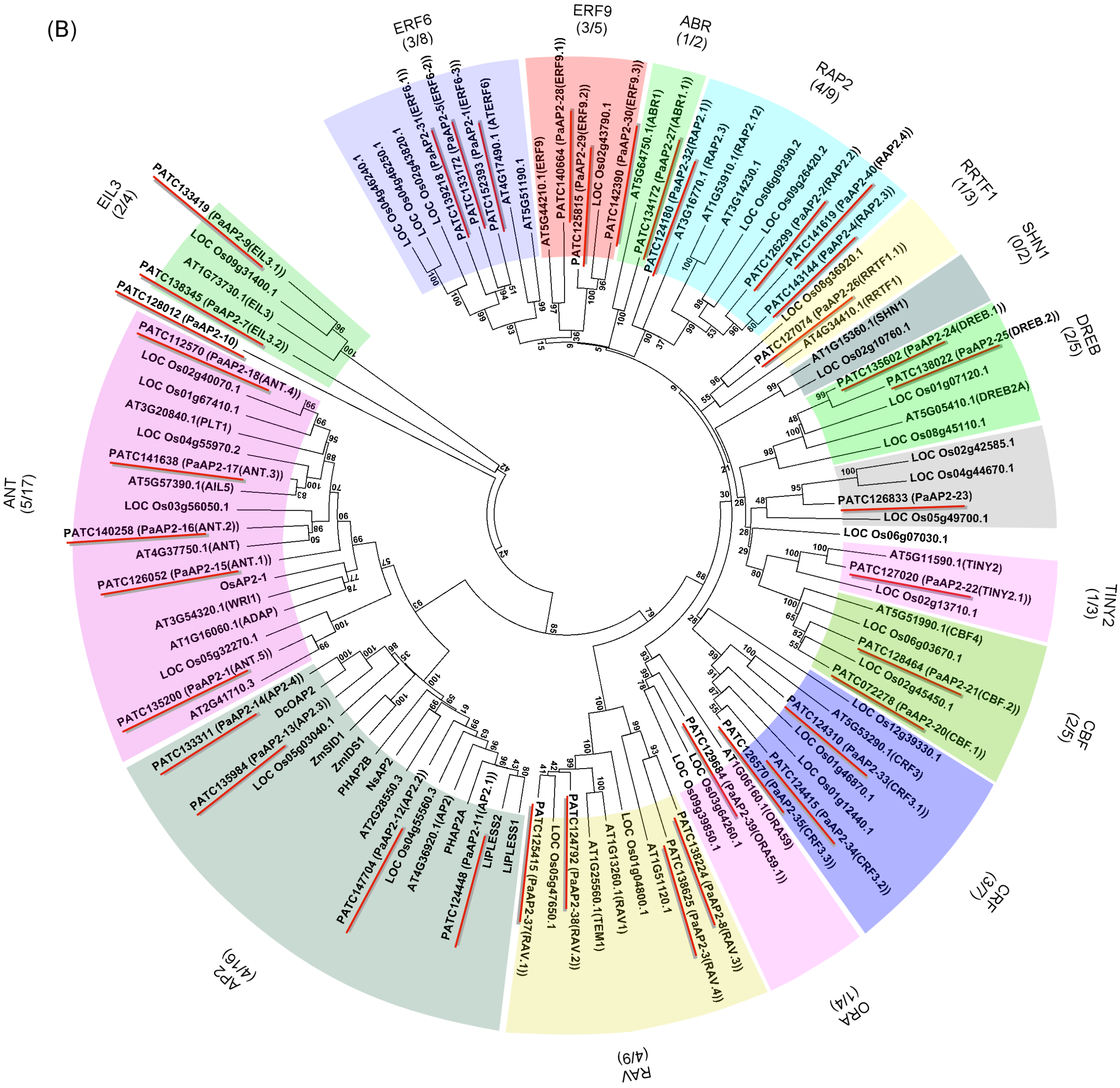

Figure S3. Phylogenetic analysis of MADS box gene and AP2 gene family. (A) Phylogenetic analysis of MADS box gene family. 30 *Arabidopsis* (AT number) and 28 rice (LOC Os number) MADS box genes were collected for phylogeny analysis and used as the mainframe to support the classification. Sequences of all other orchid species were retrieved from GenBank or related references. Initials for gene symbol are indicated as Ce (*Cymbidium ensifolium*), Dc (*Dendrobium crumenatum*), DM (*Dendrobium moniliforme*), DO (Dendrobium grex Madame Thong-IN, hybrid), Dthyr (*Dendrobium thyrsiflorum*), Phal (*Phalaenopsis* hybrid cultivar), O (*Oncidium* Gower Ramsey, hybrid), Pe (*Phalaenopsis equestris*), PATC (*Phalaenopsis aphrodite*, from Orchidstra database). (B) Phylogenetic analysis of *AP2* gene. 26 *Arabidopsis* and 33 rice *AP2* genes were used for constructing the

structure of phylogenetic analysis. Zm is the initial for *Zea mays*. *PHAP2* is petunia *AP2* homologs and *LIPLESS* is snapdragon *AP2* gene. 38 *Phalaenopsis AP2* genes were included in the analysis. Numbers in the brackets indicate number of (PATC/overall) genes within each category. A complete gene list of the phylogenetic analysis is in Supplementary Table S4.

Table S4. MADS box genes and AP2 genes in phylogenetic analysis. Altogether, there are 120 MADS box transcription factors and 105 AP2 genes applied to Supplementary Figure S3 analysis. 28 MADS box genes and 38 AP2 genes of *Phalaenopsis aphrodite* (marked with *) were denoted with PATC initials used in the *Orchidstra* database. Additional MADS box genes, 16 others, were applied to Figure 2 (panel B and C) for detailed class A and class B functional groups.

MADS box gene family

Gene Name	Accession number	Source	Reference
***Arabidopsis* (30 genes)**			
AT1G01530.1(AGL28)	NM_100035	*Arabidopsis thaliana*	Plant Cell. 2003 Jul;15(7):1538-51.
AT1G18750.1(AGL65)	NM_101733	*Arabidopsis thaliana*	Plant Physiol. 2009 Apr;149(4):1713-23.
AT1G24260.2(SEP3)	NM_001198152	*Arabidopsis thaliana*	Nature. 2000 May 11;405(6783):200-3.
AT1G26310.1(CAL)	NM_102395	*Arabidopsis thaliana*	Science. 1995 Jan 27;267(5197):522-5.
AT1G29962.1(AGL64)	NM_001084156	*Arabidopsis thaliana*	Plant Cell. 2003 Jul;15(7):1538-51.
AT1G65300.1(PHE2)	NM_105204	*Arabidopsis thaliana*	Genes Dev.2003 Jun 15;17(12):1540-53.
AT1G69120.1(AP1)	NM_105581	*Arabidopsis thaliana*	Science. 1995 Jan 27;267(5197):522-5.
AT1G69540.1(AGL94)	NM_105623	*Arabidopsis thaliana*	Plant Physiol. 2009 Apr;149(4):1713-23
AT1G77980.1(AGL66)	NM_106447	*Arabidopsis thaliana*	Plant Physiol. 2009 Apr;149(4):1713-23
AT2G03060.2(AGL30)	NM_001084404	*Arabidopsis thaliana*	Plant Physiol. 2009 Apr;149(4):1713-23
AT2G03710.1(SEP4)	NM_201682	*Arabidopsis thaliana*	Curr Biol. 2004 Nov 9;14(21):1935-40.
AT2G14210.1(ANR1)	NM_126990	*Arabidopsis thaliana*	Plant J. 2008 Jun;54(5):820-8.
AT2G22540.1(SVP)	NM_001161056	*Arabidopsis thaliana*	Plant J. 2000 Feb;21(4):351-60
AT2G42830.2(SHP2)	ABK59682	*Arabidopsis thaliana*	Nature.2000 Apr 13;404(6779):766-70.
AT2G45650.1(AGL6)	NM_130127	*Arabidopsis thaliana*	Plant Cell. 2012 Jun;24(6):2364-79.
AT2G45660.1(SOC1)	NM_130128	*Arabidopsis thaliana*	Genes Dev. 2000 Sep 15;14(18):2366-76.

AT3G02310.1(SEP2)	AEE73791	*Arabidopsis thaliana*	Curr Biol. 2004 Nov 9;14(21):1935-40.
AT3G04100.1(AGL57)	NM_111281	*Arabidopsis thaliana*	Plant Cell. 2003 Jul;15(7):1538-51.
AT3G05860.1(AGL46)	NM_111460	*Arabidopsis thaliana*	Plant Physiol. 2010 Sep;154(1):287-300.
AT3G54340.1(AP3)	AEE79216	*Arabidopsis thaliana*	Proc Natl Acad Sci U S A. 1996 May 14;93(10):4793-8.
AT3G58780.3(SHP1)	NP_001190130	*Arabidopsis thaliana*	Nature.2000 Apr 13;404(6779):766-70.
AT4G02235.1(AGL51)	AT4G02235.1	*Arabidopsis thaliana*	Plant Cell. 2003 Jul;15(7):1538-51
AT4G09960.3(STK)	AEE82818	*Arabidopsis thaliana*	Plant Cell. 2005 Mar;17(3):722-9. Epub 2005 Feb 18
AT4G18960.1(AG)	AEE84112	*Arabidopsis thaliana*	Plant Cell Physiol. 1997 Mar;38(3):248-58.
AT5G15800.2(SEP1)	NM_001125758	*Arabidopsis thaliana*	Curr Biol. 2004 Nov 9;14(21):1935-40.
AT5G20240.1(PI)	AED92817	*Arabidopsis thaliana*	Plant Cell.1991 Nov;3(11):1221-1237.
AT5G38620.1(AGL73)	NM_123223	*Arabidopsis thaliana*	J Mol Evol. 2003 May;56(5):573-86.
AT5G41200.1(AGL75)	BT030016	*Arabidopsis thaliana*	Plant Physiol.2010 Sep;154(1):287-300
AT5G48670.1(AGL80)	DQ056710	*Arabidopsis thaliana*	Plant Physiol.2010 Sep;154(1):287-300
AT5G60910(FUL)	NM_125484	*Arabidopsis thaliana*	Development. 1998 Apr;125(8):1509-17.
**Rice (28 genes)**			
LOC_Os01g10504.1_(OsMADS3)	Q40704	*Oryza sativa*	Plant Mol. Biol. 29 (1), 1-10 (1995)
LOC_Os01g18440.1_(OsMADS89)	N/A	*Oryza sativa*	BMC Genomics. 2007 Jul 18;8:242.
LOC_Os01g66030.1_(OsMADS2)	Q40702	*Oryza sativa*	Plant Sci. 109, 45-56 (1995)
LOC_Os01g66290.1_(OsMADS21)	Q8RU31	*Oryza sativa*	Plant J. 2007 Nov;52(4):690-9. Epub 2007 Sep 17
LOC_Os02g36924.1_(OsMADS27)	Q6EP49	*Oryza sativa*	Plant Cell Physiol (2003) 44 (12): 1403-1411
LOC_Os02g45770.1_(OsMADS6)	Q6EU39	*Oryza sativa*	Plant J. 2010 Nov;64(4):604-17
LOC_Os02g49840.1_(OsMADS57)	Q6Z6W2	*Oryza sativa*	BMC Genomics. 2007 Jul 18;8:242.
LOC_Os02g52340.1_(OsMADS22)	Q9XJ66	*Oryza sativa*	Mol Genet Genomics. 2005 Mar;273(1):1-9
LOC_Os03g08754.1_(OsMADS47)	Q5K4R0	*Oryza sativa*	J Exp Bot. 2008;59(8):2181-90
LOC_Os03g11614.1_(OsMADS1)	Q10PZ9	*Oryza sativa*	Plant J. 2005 Sep;43(6):915-28
LOC_Os03g54160.1_(OsMADS14)	Q10CQ1	*Oryza sativa*	Plant Mol. Biol. 44 (4), 513-527 (2000)

LOC_Os04g23910.1_(OsMADS25)	Q84NC5	*Oryza sativa*	Plant Cell Physiol. 44(12): 1403–1411 (2003)
LOC_Os04g49150.1_(OsMADS17)	Q7XUN2	*Oryza sativa*	Cell Res. 2010 Mar;20(3):299-313.
LOC_Os05g34940.1_(OsMADS4)	Q40703	*Oryza sativa*	Plant Sci. 109, 45-56 (1995)
LOC_Os06g06750.1_(OsMADS5)	Q0DEB8	*Oryza sativa*	Mol Cells. 1997 Feb 28;7(1):45-51.
LOC_Os06g11330.1_(OsMADS55)	Q69TG5	*Oryza sativa*	Plant Sci. 2012 Apr;185-186:97-104
LOC_Os06g11970.1_(OsMADS63)	N/A	*Oryza sativa*	BMC Genomics. 2007 Jul 18;8:242.
LOC_Os06g49840.1_(OsMADS16)	Q944S9	*Oryza sativa*	Plant Mol. Biol. 40 (1), 167-177 (1999)
LOC_Os07g01820.1_(OsMADS15)	Q6Q9I2	*Oryza sativa*	Plant Mol. Biol. 44 (4), 513-527 (2000)
LOC_Os07g41370.1_(OsMADS18/28)	Q0D4T4	*Oryza sativa*	Plant Physiol. 120 (4), 1193-1204 (1999)
LOC_Os08g33488.1_(OsMADS23)	Q6VAM4	*Oryza sativa*	Plant Cell Physiol (2003) 44 (12): 1403-1411.
LOC_Os08g38590.1_(OsMADS62)	N/A	*Oryza sativa*	BMC Genomics. 2007 Jul 18;8:242.
LOC_Os08g41950.1_(OsMADS7/45)	Q0J466	*Oryza sativa*	Mol Cells. 1997 Aug 31;7(4):559-66.
LOC_Os09g32948.1_(OsMADS8/24)	Q9SAR1	*Oryza sativa*	Plant J. 2010 Mar;61(5):767-81
LOC_Os10g39130.1_(OsMADS56)	P0C5B2	*Oryza sativa*	BMC Genomics. 2007 Jul 18;8:242.
LOC_Os11g43740.1_(OsMADS68)	N/A	*Oryza sativa*	BMC Genomics. 2007 Jul 18;8:242.
LOC_Os12g10540.1_(OsMADS13)	Q2QW53	*Oryza sativa*	Dev. Genet. 25 (3), 237-244 (1999)
LOC_Os12g31748.1_(OsMADS20)	Q2QQA3	*Oryza sativa*	Plant Cell Physiol (2003) 44 (12): 1403-1411
***Cymbidium* orchid (2 genes)**			
CeMADS1	GU123626	*Cymbidium ensifolium*	Plant Cell Physiol. 2011 Mar;52(3):563-77.
CeMADS2	GU123627	*Cymbidium ensifolium*	Plant Cell Physiol. 2011 Mar;52(3):563-77.
***Dendrobium* orchid (15 genes)**			
DcOAG1	DQ119840	*Dendrobium crumenatum*	Plant J. 2006 Apr;46(1):54-68.
DcOAG2	AAZ95251	*Dendrobium crumenatum*	Plant J. 2006 Apr;46(1):54-68.
DcOAP3A	AAZ95248	*Dendrobium crumenatum*	Plant J. 46 (1), 54-68 (2006)
DcOAP3B	AAZ95249	*Dendrobium crumenatum*	Plant J. 46 (1), 54-68 (2006)
DcOSEP1	DQ119842	*Dendrobium crumenatum*	Plant J. 46 (1), 54-68 (2006)

DMMADS4	ADD60473	*Dendrobium moniliforme*	Acta Hortic. 836, 259-264 (2009)
DOMADS1	AF198174	Dendrobium grex Madame Thong-In	Unpublished
DOMADS2	AF198175	Dendrobium grex Madame Thong-In	Unpublished
DOMADS3	AF198176	Dendrobium grex Madame Thong-In	Unpublished
Dthyr-PI	AAY86363	*Dendrobium thyrsiflorum*	Unpublished
DthyrAG1	DQ017702	*Dendrobium thyrsiflorum*	Gene. 2006 Feb 1;366(2):266-74.
DthyrAG2	AAY86365	*Dendrobium thyrsiflorum*	Gene. 2006 Feb 1;366(2):266-74.
DthyrFL1	AY927236	*Dendrobium thyrsiflorum*	Plant Sci. 169 (3), 579-586 (2005)
DthyrFL2	AY927237	*Dendrobium thyrsiflorum*	Plant Sci. 169 (3), 579-586 (2005)
DthyrFL3	AY927238	*Dendrobium thyrsiflorum*	Plant Sci. 169 (3), 579-586 (2005)
***Oncidium* orchid (9 genes)**			
OMADS1	HM140843	Oncidium Gower Ramsey	Plant Cell Physiol. 44 (8), 783-794 (2003)
OMADS10	HM140846	Oncidium Gower Ramsey	Plant Cell Physiol. 50 (8), 1425-1438 (2009)
OMADS11	HM140847	Oncidium Gower Ramsey	Plant Cell Physiol. 50 (8), 1425-1438 (2009)
OMADS3	AAO45824	Oncidium Gower Ramsey	Plant Cell Physiol. 43 (10), 1198-1209 (2002)
OMADS5	ADJ67234	Oncidium Gower Ramsey	Plant Physiol. 152 (2), 837-853 (2010)
OMADS6	HM140844	Oncidium Gower Ramsey	Plant Cell Physiol. 2009 Aug;50(8):1425-38.
OMADS7	HM140845	Oncidium Gower Ramsey	Plant Cell Physiol. 50 (8), 1425-1438 (2009)
OMADS8	ADJ67236	Oncidium Gower Ramsey	Plant Physiol. 152 (2), 837-853 (2010)
OMADS9	ADJ67235	Oncidium Gower Ramsey	Plant Physiol. 152 (2), 837-853 (2010)
***Phalaenopsis* orchid (37 genes)**			
PeMADS1	AF234617	*Phalaenopsis equestris*	Unpublished
PeMADS2	AAR26628	*Phalaenopsis equestris*	Plant Cell Physiol. 45 (7), 831-844 (2004)
PeMADS3	AAR26629	*Phalaenopsis equestris*	Plant Cell Physiol. 45 (7), 831-844 (2004)
PeMADS4	AAR26626	*Phalaenopsis equestris*	Plant Cell Physiol. 45 (7), 831-844 (2004)
PeMADS5	AAR26630	*Phalaenopsis equestris*	Plant Cell Physiol. 45 (7), 831-844 (2004)

PeMADS6	AAV83997	*Phalaenopsis equestris*	Plant Cell Physiol. 46 (7), 1125-1139 (2005)
PeMADS7	AFI61557	*Phalaenopsis equestris*	Plant Cell Physiol. 53 (6), 1053-1067 (2012)
PhalAG1	BAE80120	Phalaenopsis hybrid cultivar	Dev. Genes Evol. 216 (6), 301-313 (2006)
PhalAG2	BAE80121	Phalaenopsis hybrid cultivar	Dev. Genes Evol. 216 (6), 301-313 (2006)
PaAG-1	PATC052371*	*Phalaenopsis aphrodite*	*Orchidstra* database
PaAG-2	PATC138585*	*Phalaenopsis aphrodite*	*Orchidstra* database
PaAG-3	PATC155109*	*Phalaenopsis aphrodite*	*Orchidstra* database
PaAG-4	PATC202120*	*Phalaenopsis aphrodite*	*Orchidstra* database
PaAGL6-1	PATC154379*	*Phalaenopsis aphrodite*	*Orchidstra* database
PaAGL6-2	PATC138772*	*Phalaenopsis aphrodite*	*Orchidstra* database
PaANR-1	PATC129763*	*Phalaenopsis aphrodite*	*Orchidstra* database
PaANR-2	PATC131704*	*Phalaenopsis aphrodite*	*Orchidstra* database
PaAP1-1	PATC145405*	*Phalaenopsis aphrodite*	*Orchidstra* database
PaAP1-2	PATC154931*	*Phalaenopsis aphrodite*	*Orchidstra* database
PaAP3-1	PATC240636*	*Phalaenopsis aphrodite*	*Orchidstra* database
PaAP3-2	PATC133864*	*Phalaenopsis aphrodite*	*Orchidstra* database
PaAP3-3	PATC138350*	*Phalaenopsis aphrodite*	*Orchidstra* database
PaAP3-4	PATC154853*	*Phalaenopsis aphrodite*	*Orchidstra* database
PaMdeltaP-1	PATC132082*	*Phalaenopsis aphrodite*	*Orchidstra* database
PaMdeltaS-1	PATC138798*	*Phalaenopsis aphrodite*	*Orchidstra* database
PaMgamma-1	PATC171265*	*Phalaenopsis aphrodite*	*Orchidstra* database
PaMgamma-2	PATC240637*	*Phalaenopsis aphrodite*	*Orchidstra* database
PaMgamma-3	PATC240639*	*Phalaenopsis aphrodite*	*Orchidstra* database
PaMgamma-4	PATC240638*	*Phalaenopsis aphrodite*	*Orchidstra* database
PaPI-1	PATC152852*	*Phalaenopsis aphrodite*	*Orchidstra* database
PaSEP-1	PATC138540*	*Phalaenopsis aphrodite*	*Orchidstra* database

PaSEP-2	PATC141808*	*Phalaenopsis aphrodite*	*Orchidstra* database
PaSEP-3	PATC152066*	*Phalaenopsis aphrodite*	*Orchidstra* database
PaSOC1-1	PATC136427*	*Phalaenopsis aphrodite*	*Orchidstra* database
PaSOC1-2	PATC150808*	*Phalaenopsis aphrodite*	*Orchidstra* database
PaSOC1-3	PATC154491*	*Phalaenopsis aphrodite*	*Orchidstra* database
PaSVP-1	PATC127095*	*Phalaenopsis aphrodite*	*Orchidstra* database
**Others (16 genes)**			
LMADS1	AAM27456	*Lilium longiflorum*	Plant Cell Physiol. 42 (10), 1156-1168 (2001)
TGEFA	BAC75970	*Tulipa gesneriana*	Plant Mol. Biol. 52 (4), 831-841 (2003)
BOBAP3	AAB17139	*Brassica oleracea var. botrytis*	Planta 201 (2), 179-188 (1997)
PMADS1	Q07472	Petunia x hybrida	Plant Physiol. 102 (3), 1051-1052 (1993)
ZMM16	NP_001105136	*Zea mays*	Science 326 (5956), 1112-1115 (2009)
ZMM29	NP_001105137	*Zea mays*	Plant Physiol. 134 (4), 1317-1326 (2004)
LRGLOA	BAB91551	*Lilium regale*	Mol. Biol. Evol. 19 (5), 587-596 (2002)
LRGLOB	BAB91552	*Lilium regale*	Mol. Biol. Evol. 19 (5), 587-596 (2002)
ORCPI	BAC22579	*Orchis italica*	Unpublished
VvAP1	ACZ26528	*Vitis vinifera*	Unpublished
LlAP1	ADT78583	*Lilium longiflorum*	Plant Cell Physiol. 49 (5), 704-717 (2008)
ZmAP1	NP_001105333	*Zea mays*	Plant J. 8 (6), 845-854 (1995)
MgAGL6	AAY25579	*Magnolia grandiflora*	Unpublished
AcAGL6	AFX72880	*Aquilegia coerulea*	Unpublished
GmAGL6	XP_003528894	*Glycine max*	Unpublished
TaAGL6	ABF57913	*Triticum aestivum*	Mol. Genet. Genomics 276 (4), 334-350 (2006)

AP2 gene family

Gene Name	Accession number	Source	Reference

Arabidopsis (26 genes)		
AT1G06160.1(ORA59)	*Arabidopsis thaliana*	http://www.arabidopsis.org/
AT1G13260.1(RAV1)	*Arabidopsis thaliana*	Nucleic Acids Res. 1999 Jan 15;27(2):470-8
AT1G15360.1(SHN1)	*Arabidopsis thaliana*	http://www.arabidopsis.org/
AT1G16060.1(ADAP)	*Arabidopsis thaliana*	http://www.arabidopsis.org/
AT1G25560.1(TEM1)	*Arabidopsis thaliana*	http://www.arabidopsis.org/
AT1G51120.1	*Arabidopsis thaliana*	http://www.arabidopsis.org/
AT1G53910.1(RAP2.12)	*Arabidopsis thaliana*	Proc Natl Acad Sci U S A. 1997 Jun 24;94(13):7076-81
AT1G73730.1(EIL3)	*Arabidopsis thaliana*	J Mol Biol. 2005 Apr 29;348(2):253-64
AT2G28550.3	*Arabidopsis thaliana*	http://www.arabidopsis.org/
AT2G41710.3	*Arabidopsis thaliana*	http://www.arabidopsis.org/
AT3G14230.1	*Arabidopsis thaliana*	http://www.arabidopsis.org/
AT3G16770.1_(RAP2.3)	*Arabidopsis thaliana*	http://www.arabidopsis.org/
AT3G20840.1(PLT1)	*Arabidopsis thaliana*	http://www.arabidopsis.org/
AT3G54320.1(WRI1)	*Arabidopsis thaliana*	http://www.arabidopsis.org/
AT4G17490.1_(ATERF6)	*Arabidopsis thaliana*	http://www.arabidopsis.org/
AT4G34410.1(RRTF1)	*Arabidopsis thaliana*	http://www.arabidopsis.org/
AT4G36920.1(AP2)	*Arabidopsis thaliana*	Plant Cell. 1989 Jan;1(1):37-52
AT4G37750.1(ANT)	*Arabidopsis thaliana*	Sex Plant Reprod. 2010 Jun;23(2):115-21
AT5G05410.1(DREB2A)	*Arabidopsis thaliana*	Plant Cell. 1998 Aug;10(8):1391-406
AT5G11590.1(TINY2)	*Arabidopsis thaliana*	http://www.arabidopsis.org/
AT5G44210.1(ERF9)	*Arabidopsis thaliana*	http://www.arabidopsis.org/
AT5G51190.1	*Arabidopsis thaliana*	http://www.arabidopsis.org/
AT5G51990.1(CBF4)	*Arabidopsis thaliana*	http://www.arabidopsis.org/
AT5G53290.1(CRF3)	*Arabidopsis thaliana*	http://www.arabidopsis.org/
AT5G57390.1(AIL5)	*Arabidopsis thaliana*	http://www.arabidopsis.org/

AT5G64750.1(ABR1)	*Arabidopsis thaliana*	http://www.arabidopsis.org/
**Rice (33 genes)**		
LOC_Os01g04800.1	*Oryza sativa*	http://rice.plantbiology.msu.edu/
LOC_Os01g07120.1	*Oryza sativa*	http://rice.plantbiology.msu.edu/
LOC_Os01g12440.1	*Oryza sativa*	http://rice.plantbiology.msu.edu/
LOC_Os01g46870.1	*Oryza sativa*	http://rice.plantbiology.msu.edu/
LOC_Os01g67410.1	*Oryza sativa*	http://rice.plantbiology.msu.edu/
LOC_Os02g10760.1	*Oryza sativa*	http://rice.plantbiology.msu.edu/
LOC_Os02g13710.1	*Oryza sativa*	http://rice.plantbiology.msu.edu/
LOC_Os02g40070.1	*Oryza sativa*	http://rice.plantbiology.msu.edu/
LOC_Os02g42585.1	*Oryza sativa*	http://rice.plantbiology.msu.edu/
LOC_Os02g43790.1	*Oryza sativa*	http://rice.plantbiology.msu.edu/
LOC_Os02g43820.1	*Oryza sativa*	http://rice.plantbiology.msu.edu/
LOC_Os02g45450.1	*Oryza sativa*	http://rice.plantbiology.msu.edu/
LOC_Os03g56050.1	*Oryza sativa*	http://rice.plantbiology.msu.edu/
LOC_Os03g64260.1	*Oryza sativa*	http://rice.plantbiology.msu.edu/
LOC_Os04g44670.1	*Oryza sativa*	http://rice.plantbiology.msu.edu/
LOC_Os04g46240.1	*Oryza sativa*	http://rice.plantbiology.msu.edu/
LOC_Os04g46250.1	*Oryza sativa*	http://rice.plantbiology.msu.edu/
LOC_Os04g55560.3	*Oryza sativa*	http://rice.plantbiology.msu.edu/
LOC_Os04g55970.2	*Oryza sativa*	http://rice.plantbiology.msu.edu/
LOC_Os05g03040.1	*Oryza sativa*	http://rice.plantbiology.msu.edu/
LOC_Os05g32270.1	*Oryza sativa*	http://rice.plantbiology.msu.edu/
LOC_Os05g47650.1	*Oryza sativa*	http://rice.plantbiology.msu.edu/
LOC_Os05g49700.1	*Oryza sativa*	http://rice.plantbiology.msu.edu/
LOC_Os06g03670.1	*Oryza sativa*	http://rice.plantbiology.msu.edu/

LOC_Os06g07030.1		*Oryza sativa*	http://rice.plantbiology.msu.edu/
LOC_Os06g09390.2		*Oryza sativa*	http://rice.plantbiology.msu.edu/
LOC_Os08g36920.1		*Oryza sativa*	http://rice.plantbiology.msu.edu/
LOC_Os08g45110.1		*Oryza sativa*	http://rice.plantbiology.msu.edu/
LOC_Os09g26420.2		*Oryza sativa*	http://rice.plantbiology.msu.edu/
LOC_Os09g31400.1		*Oryza sativa*	http://rice.plantbiology.msu.edu/
LOC_Os09g39850.1		*Oryza sativa*	http://rice.plantbiology.msu.edu/
LOC_Os12g39330.1		*Oryza sativa*	http://rice.plantbiology.msu.edu/
OsAP2-1	AB247626	*Oryza sativa*	Sex Plant Reprod (2006)19:197-206
***Phalaenopsis* orchid (38 genes)**			
PaAP2-20(CBF.1)	PATC072278*	*Phalaenopsis aphrodite*	*Orchidstra* database
PaAP2-18(ANT.4)	PATC112570*	*Phalaenopsis aphrodite*	*Orchidstra* database
PaAP2-32(RAP2.1)	PATC124180*	*Phalaenopsis aphrodite*	*Orchidstra* database
PaAP2-33(CRF3.1)	PATC124310*	*Phalaenopsis aphrodite*	*Orchidstra* database
PaAP2-34(CRF3.2)	PATC124415*	*Phalaenopsis aphrodite*	*Orchidstra* database
PaAP2-11(AP2.1)	PATC124448*	*Phalaenopsis aphrodite*	*Orchidstra* database
PaAP2-38(RAV.2)	PATC124792*	*Phalaenopsis aphrodite*	*Orchidstra* database
PaAP2-37(RAV.1)	PATC125415*	*Phalaenopsis aphrodite*	*Orchidstra* database
PaAP2-29(ERF9.2)	PATC125815*	*Phalaenopsis aphrodite*	*Orchidstra* database
PaAP2-15(ANT.1)	PATC126052*	*Phalaenopsis aphrodite*	*Orchidstra* database
PaAP2-2(RAP2.2)	PATC126299*	*Phalaenopsis aphrodite*	*Orchidstra* database
PaAP2-35(CRF3.3)	PATC126570*	*Phalaenopsis aphrodite*	*Orchidstra* database
PaAP2-23	PATC126833*	*Phalaenopsis aphrodite*	*Orchidstra* database
PaAP2-22(TINY2.1)	PATC127020*	*Phalaenopsis aphrodite*	*Orchidstra* database
PaAP2-26(RRTF1.1)	PATC127074*	*Phalaenopsis aphrodite*	*Orchidstra* database
PaAP2-10	PATC128012*	*Phalaenopsis aphrodite*	*Orchidstra* database

PaAP2-21(CBF.2)	PATC128464*	*Phalaenopsis aphrodite*	*Orchidstra* database
PaAP2-39(ORA59.1)	PATC129684*	*Phalaenopsis aphrodite*	*Orchidstra* database
PaAP2-5(ERF6-2)	PATC133172*	*Phalaenopsis aphrodite*	*Orchidstra* database
PaAP2-14(AP2-4)	PATC133311*	*Phalaenopsis aphrodite*	*Orchidstra* database
PaAP2-9(EIL3.1)	PATC133419*	*Phalaenopsis aphrodite*	*Orchidstra* database
PaAP2-27(ABR1.1)	PATC134172*	*Phalaenopsis aphrodite*	*Orchidstra* database
PaAP2-1(ANT.5)	PATC135200*	*Phalaenopsis aphrodite*	*Orchidstra* database
PaAP2-24(DREB.1)	PATC135602*	*Phalaenopsis aphrodite*	*Orchidstra* database
PaAP2-13(AP2.3)	PATC135984*	*Phalaenopsis aphrodite*	*Orchidstra* database
PaAP2-25(DREB.2)	PATC138022*	*Phalaenopsis aphrodite*	*Orchidstra* database
PaAP2-8(RAV.3)	PATC138224*	*Phalaenopsis aphrodite*	*Orchidstra* database
PaAP2-7(EIL3.2)	PATC138345*	*Phalaenopsis aphrodite*	*Orchidstra* database
PaAP2-3(RAV.4)	PATC138625*	*Phalaenopsis aphrodite*	*Orchidstra* database
PaAP2-31(ERF6.1)	PATC139218*	*Phalaenopsis aphrodite*	*Orchidstra* database
PaAP2-16(ANT.2)	PATC140258*	*Phalaenopsis aphrodite*	*Orchidstra* database
PaAP2-28(ERF9.1)	PATC140664*	*Phalaenopsis aphrodite*	*Orchidstra* database
PaAP2-40(RAP2.4)	PATC141619*	*Phalaenopsis aphrodite*	*Orchidstra* database
PaAP2-17(ANT.3)	PATC141638*	*Phalaenopsis aphrodite*	*Orchidstra* database
PaAP2-30(ERF9.3)	PATC142390*	*Phalaenopsis aphrodite*	*Orchidstra* database
PaAP2-4(RAP2.3)	PATC143144*	*Phalaenopsis aphrodite*	*Orchidstra* database
PaAP2-12(AP2.2)	PATC147704*	*Phalaenopsis aphrodite*	*Orchidstra* database
PaAP2-1(ERF6-3)	PATC152393*	*Phalaenopsis aphrodite*	*Orchidstra* database
**Others (8 genes)**			
PHAP2A	AAD39439	Petunia x hybrida	Plant Cell. 2001 Feb;13(2):229-44.
PHAP2B	AAD39440	Petunia x hybrida	Plant Cell. 2001 Feb;13(2):229-45.
ZmIDS1	NP_001104904	*Zea mays*	Development 135 (18), 3013-3019 (2008)

ZmSID1	NP_001139539	*Zea mays*	Development 135 (18), 3013-3019 (2009)
DcOAP2	AAZ95247	*Dendrobium crumenatum*	Plant J. 46 (1), 54-68 (2006)
LIPLESS1	AAO52746	*Antirrhinum majus*	EMBO J. 22 (5), 1058-1066 (2003)
LIPLESS2	AAO52747	*Antirrhinum majus*	EMBO J. 22 (5), 1058-1066 (2004)
NsAP2	BAL04981	Nymphaea hybrid cultivar	Plant Cell Rep. 30 (10), 1909-1918 (2011)

Subcellular localization of *Phalaenopsis aphrodite* MADS box genes

Method

GFP fusion constructs were prepared followed by particle bombardment and confocal microscopy with the same procedures on previous report [2]. Full length cDNA of target genes were PCR amplified and cloned into a smGFP vector (326-GFP) [3]. Nuclei marker construct of mCherry with NLS signal peptide fusion (E3170) was kindly provided by Dr. Gelvin (Purdue University, IN) [4]. The *GFP* construct and nucleus mCherry construct were co-bombarded into the petal of *Phalaenopsis* Sogo Yukidian 'V3' (a commercial hybrid) for further observation with confocal microscopy and image taken (Supplementary Figure S3). Free GFP construct was also bombarded to orchid petals and showed diffused cytoplasmic pattern (data not shown) as described before [2]. All transient expression experiments were repeated at least three times of bombardments and more than three images were taken from each bombardment.

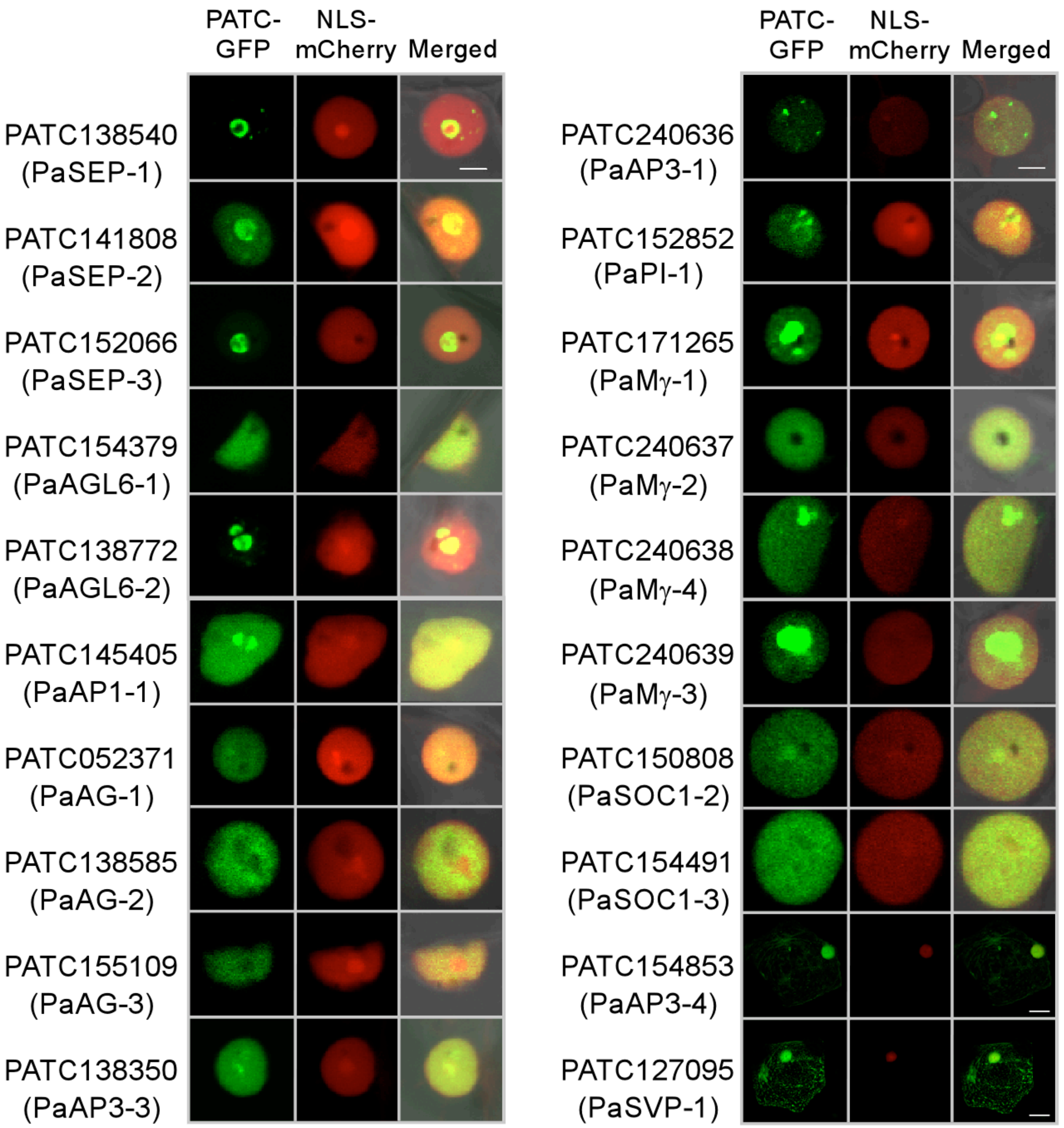

Figure S4. Subcellular localization of MADS box genes according to particle bombardment. All *GFP* fusion patterns exhibit nuclei localization and co-localized with nuclear marker of NLS-mCherry except for *PaAP3-4* (PATC154853) and *PaSVP-1* (PATC127095), showing both nuclei and cytoplasmic punctured pattern. (Scale bars in the photo of nuclei pattern indicate 5μm; and bars in *PaAP3-4* and *PaSVP-1* indicate 20μm.)

Morphology of the peloric mutants

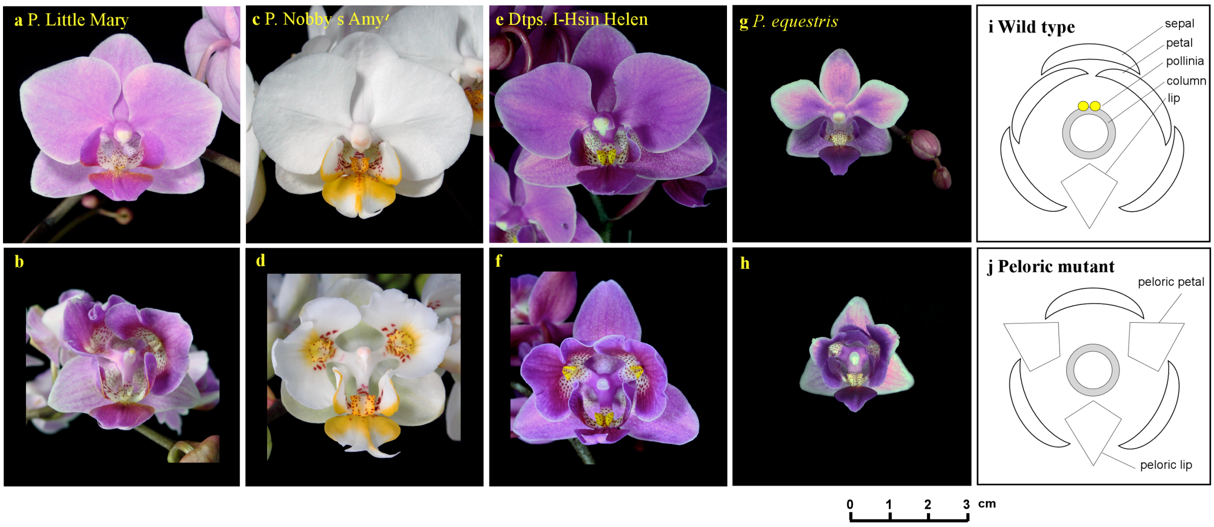

Figure S5. Peloric flowers of orchids. Peloric mutants can be found in several orchid cultivars including commercial hybrids. P. Little Mary and P. Nobby's Amy are commercial hybrids of crossing between *Phalaenopsis spp*. Dtps stands for *Doritaenopsis* and is a commercial hybrid of crossing between *Doritis* and *Phalaenopsis* orchids. Box a and b are *Phalaenopsis* Little Mary; c and d are *Phalaenopsis* Nobby's Amy; e and f are Dtps I-Hsing Helen; g and h are *Phalaenopsis equestris*. Box a, c, e and g are wild type flowers. Box b, d, f and h are peloric flowers. Box i and j are illustrations of the morphology of wild type and peloric flowers

Reference

1. Su CL, Chao YT, Yen SH, Chen CY, Chen WC, et al. (2013) Orchidstra: an integrated orchid functional genomics database. Plant Cell Physiol 54: e11.
2. Su CL, Chao YT, Alex Chang YC, Chen WC, Chen CY, et al. (2011) De novo assembly of expressed transcripts and global analysis of the Phalaenopsis aphrodite transcriptome. Plant Cell Physiol 52: 1501-1514.
3. Davis SJ, Vierstra RD (1998) Soluble, highly fluorescent variants of green fluorescent protein (GFP) for use in higher plants. Plant Mol Biol 36: 521-528.
4. Lee LY, Fang MJ, Kuang LY, Gelvin SB (2008) Vectors for multi-color bimolecular fluorescence complementation to investigate protein-protein interactions in living plant cells. Plant Methods 4: 24.
